# Interpretable model using serum biomarkers and intra-/peri-aneurysmal CTA radiomics for discriminating ruptured from unruptured intracranial aneurysms: an external validation retrospective study

**DOI:** 10.3389/fneur.2026.1867021

**Published:** 2026-10-07

**Authors:** Juan Liu, Zhipeng Chen, Kun Wang, Chunyue Yan, Fei Wang, Xiaolei Sun

**Affiliations:** 1 Department of Interventional Medicine, The Affiliated Hospital of Southwest Medical University, Luzhou, China; 2 Department of Radiology, Luzhou People's Hospital, Luzhou, China; 3 Department of Emergency Medicine, Luzhou People's Hospital, Luzhou, China; 4 The Fourth People’s Hospital of Sichuan Province, West China Chunxi Hospital of Sichuan University, Chengdu, Sichuan, China; 5 Department of General Surgery (Vascular Surgery), The Affiliated Hospital of Southwest Medical University, Luzhou, Sichuan, China

**Keywords:** CTA, intracranial aneurysms, machine learning, radiomics, rupture, SHapley Additive exPlanations

## Abstract

**Objective:**

To develop an interpretable model that integrates diverse machine learning algorithms, fusion strategies, intra−/perianeurysmal CTA radiomics, and serum biomarkers to differentiate ruptured from unruptured intracranial aneurysms (IAs), and to investigate the underlying biological features associated with rupture status.

**Methods:**

Totally, 744 IAs patients who underwent CTA were retrospectively enrolled. Data from Center 1 (*N* = 423) served as the training set, and data from Center 2 (*N* = 321) served as the external validation set. Volumes of interest were delineated in intra-aneurysmal and peri-aneurysmal 1 mm regions. The serum inflammation score (SIS) was constructed using serum biomarkers. Seven machine learning classifiers were employed to develop models, and the optimal fusion model was developed by combining all classifiers using arithmetic mean, geometric mean, weighted mean, and logistic regression (LRSLM). Model performance was assessed by the area under the curve (AUC) and decision curve analysis (DCA), while interpretability was evaluated via SHAP, correlation, and restricted cubic spline (RCS) analyses.

**Results:**

Among the 11 models, LRSLM exhibited robust performance in the external validation set, achieving an AUC of 0.9177 with good calibration. It also demonstrated high accuracy (0.8847), recall (0.8171), and a low Brier score (0.0999), alongside greater clinical net benefit across a wide range of threshold probabilities (10–85%). Correlation analyses revealed strong positive associations between intra-aneurysmal and peri-aneurysmal radiomic features (Spearman’s *r* = 0.62, 0.72, and 0.84, respectively; all *p* < 0.001), as well as significant positive correlations between the intra−/perianeurysmal radiomics and SIS (Spearman’s *r* = 0.32, 0.29, 0.32, and 0.34; all *p* < 0.001). RCS analysis further demonstrated a significant non-linear relationship between the intra-aneurysmal (IRSIAs) and peri-aneurysmal (PRSIAs) radiomic features and an increased incidence of IAs rupture (Non-linear *p* < 0.05; Overall *p* < 0.05). SHAP analysis highlighted key radiomics features contributors to the fusion model and supported model interpretability.

**Conclusion:**

The LRSLM model achieves robust discriminative performance for differentiating ruptured from unruptured IAs. SHAP analysis enhances the interpretability of the model and may assist in identifying high-risk phenotypic features, although prospective validation is required before predictive clinical application can be considered.

## 1 Introduction

In recent years, with the widespread adoption of CTA scanning technology, the detection rate of intracranial aneurysms (IAs) has significantly increased (1). As a potentially fatal disease, IAs have an adult prevalence rate of approximately 1 to 3% (2). Once the aneurysm ruptures, leading to subarachnoid hemorrhage (aSAH), it results in extremely high mortality and morbidity rates (2–4). Additionally, patients with unruptured IAs often experience psychological issues such as anxiety and depression, with incidence rates reaching as high as 28 and 21%, respectively (5), posing severe threats to their health and imposing substantial medical and economic burdens. Therefore, early, precise, and timely assessment of IAs rupture status is crucial for guiding clinical intervention.

Radiomics extracts quantitative features from medical images through high-throughput methods, enabling the quantification of lesion heterogeneity information that conventional imaging cannot capture. In recent years, multiple studies have demonstrated the potential value of CTA radiomics in differentiating ruptured from unruptured IAs (6–12). However, while most studies have indicated that radiomics can effectively enhance the predictive efficacy of IAs rupture risk, some research suggests that combining radiomics with morphological parameters of IAs does not show superior predictive capabilities and notes that CTA-based radiomics models are not better than traditional morphological feature models (6–8). Furthermore, these studies corroborate the association between aneurysm hemodynamic changes and rupture from a radiomics perspective. Yet, existing research primarily focuses on the radiomics analysis of the aneurysm lumen itself, while aneurysm rupture is influenced not only by hemodynamics but also closely related to the structural integrity of the aneurysm wall and the stimulation of the surrounding inflammatory microenvironment. Moreover, Radiomics features based solely on the aneurysm lumen are insufficient to comprehensively reflect the pathophysiological changes in the aneurysm wall and adjacent tissues. Theoretically, extending the CTA aneurysm region of interest to include the perilesional tissue (e.g., a certain range beyond the lumen) may capture imaging phenotypic information related to aneurysm wall inflammation and microcirculatory changes, thereby providing additional predictive value. Based on this, this study aims to explore the value of radiomics features from a CTA region expanded by 1 mm beyond the aneurysm lumen in differentiating the rupture status of IAs, assessing whether it can supplement the IAs radiomics model to provide more comprehensive evidence for the non-invasive clinical assessment of aneurysm rupture status.

Importantly, inflammatory responses play a pivotal role in the initiation, progression, and rupture of IAs. Previous studies have shown that inflammatory cell infiltration, endothelial dysfunction, and extracellular matrix degradation in the aneurysm wall are the core mechanisms leading to structural weakening and subsequent rupture (13–15). Inflammation is crucial in the structural damage of IAs walls (14), and the ruptured IAs walls often exhibit inflammatory cell infiltration, whereas unruptured IAs walls rarely do (16). Serum inflammatory biomarkers, such as the neutrophil-to-lymphocyte ratio (NLR), reflect the overall systemic inflammatory burden and offer advantages like ease of acquisition and strong reproducibility, making them a focus in recent years for IA risk assessment. Zheng et al. (17) found that NLR was independently associated with symptomatic unruptured aneurysms, with an optimal cutoff value of 2.38. Moreover, Wu et al. (18) further reported that NLR levels were significantly higher in patients with ruptured aneurysms compared to the unruptured group (7.55 *vs*. 1.81; *p* < 0.001). These findings suggest that serum inflammatory biomarkers may serve as indirect indicators of aneurysm wall inflammatory burden and hold potential value in risk stratification for rupture. However, current research may lack systematic evaluation of the combined predictive efficacy of different inflammatory biomarkers, and it remains unclear whether serum inflammatory biomarkers provide supplementary value to CTA radiomics for differentiating ruptured from unruptured IAs.

Machine learning algorithms, including Bayes, decision tree (DecisionTree), k-nearest neighbors (KNN), logistic regression (LR), artificial neural networks (NNet), support vector machines (SVM), and extreme gradient boosting classifiers (XGBoost) can handle high-dimensional, nonlinear data and uncover potential patterns that are difficult to identify through conventional statistical analysis. Recent studies have combined radiomics features with machine learning for predicting the risk of IAs rupture, demonstrating promising application prospects (19–23). However, existing models of predicting IAs rupture still have certain limitations. Firstly, most studies only based on CTA imaging and radiomics features, ignoring the biological information of systemic inflammatory response, which is closely related to the inflammation and progression of arterial aneurysm walls. Secondly, there is a lack of fusion strategies on the basis of existing traditional machine learning algorithms. Moreover, current machine learning models mostly belong to the “black box” mode, lacking interpretability analysis and external verification of prediction results.

Therefore, this study aims to compare and develop an interpretable model for differentiating ruptured from unruptured IAs using different machine learning algorithms, intra-aneurysmal and peri-aneurysmal 1 mm CTA-radiomics and serum biomarkers. Moreover, we further sought to explore the relationships among these multi-source features and to identify the most influential contributors to the classification decision using SHapley Additive exPlanations (SHAP) analysis.

## 2 Materials and methods

### 2.1 Patients, inclusion and exclusion criteria

This study was approved by the Ethics Committee of Luzhou People’s Hospital (LLW202401012) and The Affiliated Hospital of Southwest Medical University (KY2025378). Given the retrospective design, the requirement for informed consent was waived, and all patient data were anonymized before analysis.

*Inclusion criteria*: (1) Age > 18 years, with complete cranial CTA images; (2) Single aneurysm; (3) Complete blood routine and biochemical tests were performed upon admission.

*Exclusion criteria*: (1) non-saccular IAs (such as fusiform and serpentine IAs, dissecting IAs, or pseudoaneurysms); (2) IAs combined with other vascular diseases; (3) IAs size < 2 mm; intracranial aneurysms that could not be differentiated from infundibulum on CTA images; (4) Poor quality of CTA images; (5) IAs surgery or interventional treatment had been performed before CTA examination; (6) History of trauma, fever or acute infection within 2 weeks before admission; (7) Unclear of IAs rupture status.

According to previous literature (22, 24), IAs were classified into the ruptured group and the unruptured group. The criteria for IAs rupture are as follows: For patients with subarachnoid hemorrhage, when the aneurysm adjacent to the cisternal clots was identified with CTA, the aneurysm was judged to be ruptured; if the aneurysm is found not located near the blood clot, its rupture status will be determined during surgery. Aneurysms without symptoms of subarachnoid hemorrhage or other symptoms are classified as unruptured aneurysms.

Between January 2018 and December 2023, 423 IAs from Luzhou People’s Hospital were collected as the training set, and 321 IAs from The Affiliated Hospital of Southwest Medical University were collected as the external validation set from January 2023 to December 2024. Supplementary Figure 1 shows the flowchart of patient screening. Ultimately, 744 IAs patients were analyzed in this study.

### 2.2 Baseline information and CTA scanning techniques

Patient demographic and clinical laboratory data, including age, white blood cells (WBC), neutrophils (Neu), monocytes (MO), platelets (PLT), alanine aminotransferase (ALT), alkaline phosphatase (ALP), gamma-glutamyl transferase (GGT), etc., were collected. Importantly, 16 inflammatory-derived indicators were definition and calculated, and the definition formulas were summarized in Supplementary Table 1. Feature selection was performed using the least absolute shrinkage and selection operator (LASSO) regression to identify optimal serum biomarkers, and the serum inflammation score (SIS) was constructed by logistic regression (25).

All IAs patients underwent CTA scans using GE Revolution 256 CT from Luzhou People’s Hospital (*N* = 423), and Philips Brilliance iCT256 (*N* = 280) and uCT780 (*N* = 41) from The Affiliated Hospital of Southwest Medical University. The detailed scanning parameters are summarized in Supplementary Table 2.

### 2.3 CTA preprocessing and IAs segmentation

Before exporting the CTA images from the PACS system, we masked all the personal information of the IAs patients (name, phone number and address), and re-named each patient using a numerical format. Image preprocessing was essential to standardize radiomics feature extraction. Prior to segmentation, all CTA images were resampled to a uniform voxel size of 1 mm × 1 mm × 1 mm to minimize variability arising from different imaging devices and acquisition parameters. In addition, intensity normalization and gray-level discretization (bin width = 25) were applied to further standardize the CTA images.

IAs segmentation was performed using 3D Slicer (version 5.0.2, https://www.slicer.org/). Reader 1 and Reader 2 (J.L. and F.W., with 6 and 12 years of experience in CT imaging, respectively) manually delineated the IAs on the preprocessed CTA images to generate three-dimensional volumes of interest (VOIs). The 1 mm peri-aneurysmal region extending from the boundary of each IAs was automatically generated using the “Margin” module in 3D Slicer, and adjacent blood vessels, clearly identifiable extravasated blood adjacent to the IAs, and brain tissue were manually removed. Ultimately, three distinct VOIs were defined: intra-aneurysm, peri-aneurysmal 1 mm, and intra-perianeurysmal 1 mm. To assess feature consistency, a subset of 50 randomly selected IAs patients was independently re-segmented by Reader 1 and Reader 3 (K.W., with 10 years of of experience in CT imaging) after a one-month interval. Using the correlation coefficients (ICCs) of Readers 1 and 2, Readers 1 and 3, and the previous and subsequent sections of Reader 1, three sets of ICCs were employed to evaluate the repeatability and stability of the extracted radiomics features. Only the radiomics features with three sets of ICCs greater than 0.8 were used for the subsequent analysis.

### 2.4 Radiomics feature extraction and dimensionality reduction

Radiomic features were extracted using the PyRadiomics package, yielding 851 features from each of the three VOIs, for a total of 2,553 features. Importantly, to prevent data leakage, all feature selection processes and model construction were carried out solely on the training set. Throughout the entire model development and feature selection process, the data from the external validation set remains “invisible.”

In the training set, only features with an ICC > 0.80 were retained following normalization. Dimensionality reduction was then performed through a sequential process: (a) screening for group differences using t-tests or Mann–Whitney *U* test (*p* ≤ 0.05); (b) removing highly correlated features (Spearman’s correlation > 0.9); (c) applying maximum relevance minimum redundancy (mRMR) selection; (d) ranking the remaining features using the XGBoost algorithm and retaining the top 15 based on SHapley Additive exPlanations (SHAP) values (26); and (e) performing feature selection using the least absolute shrinkage and selection operator (LASSO) with 10-fold cross-validation. Finally, based on the non-zero coefficients and weights of the residual features, the radiomics scores of intra-aneurysm (IRSIAs), peri-aneurysmal 1 mm (PRSIAs), and intra-perianeurysmal 1 mm (IPRSIAs) were calculated for IAs patients. Of note, we previously applied a similar radiomic fusion framework to predict microvascular invasion in hepatocellular carcinoma (27), which informed the methodological design of the present study. The combined Radiomics Score (CRS) of three VOIs was constructed by logistic regression.

### 2.5 Model building and evaluation

In the training set, the univariate and multivariate logistic regression were used to identify independent risk factors. The independent risk factors that had a *p* < 0.05 were utilized for the subsequent development of models. Ultimately, seven machine learning models of Bayes, DecisionTree, KNN, LRLASSO, NNet, SVM, and XGBoost were built in the training set. Importantly, we conducted a systematic hyperparameter optimization for seven machine learning models, mainly employing the methods of grid search and 10-fold cross-validation. The detailed hyperparameter optimization parameters are summarized in Supplementary Table 3. The optimal model was integrated for all machine learning using arithmetic mean (aMSLM), geometric mean (gMSLM), weighted mean (wMSLM), and logistic regression (LRSLM).

Model performance was assessed using the area under the curve (AUC), accuracy, precision, recall, F1 score, and Brier score. Calibration was evaluated using calibration curves, and clinical utility was assessed via decision curve analysis (DCA). The imbalanced data of the categories are analyzed using the balanced accuracy and the precision-recall AUC (PR-AUC).

### 2.6 Model interpretability analysis

To assess the association and agreement between intra-aneurysm and peri-aneurysmal 1 mm, the Correlation analysis and Restrictive cubic spline analysis (RCS) were performed. The SHAP framework elucidated model interpretability via global feature rankings and force plots, enabling biological and clinical insights. The detailed design and process are shown in Figure 1.

**Figure 1:**
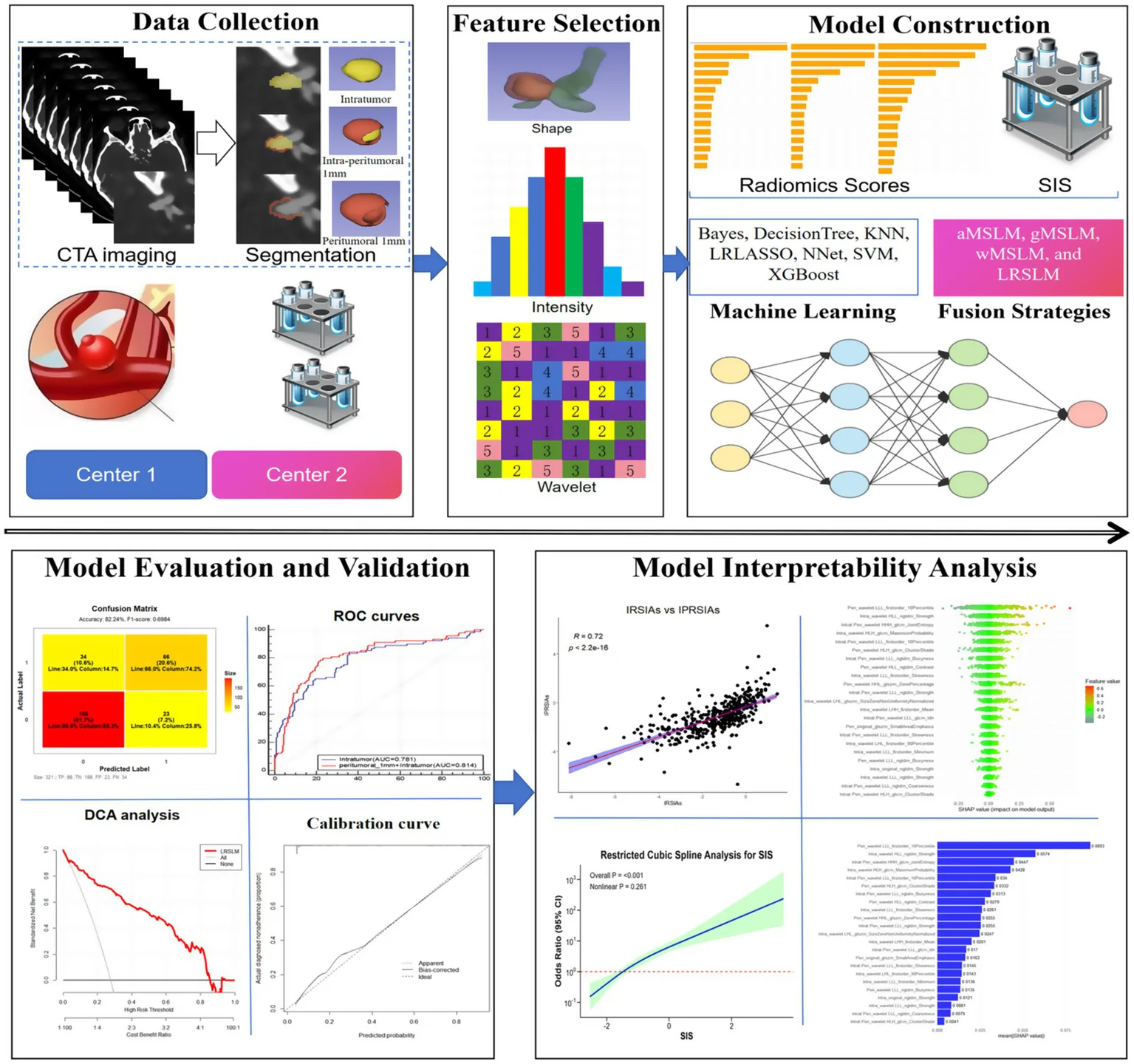
The design and workflow of this study.

### 2.7 Statistical analysis

All statistical analyses and model construction were conducted using R software (version 4.1.2, http://www.r-project.org/) and MedCalc (version 15.2). Machine learning classifiers were developed using the R packages “mlr3,” “glm,” “rms,” “glmnet,” “caret,” “kNN,” “e1071,” “randomForest,” “xgboost,” “lightgbm,” “gbm,” and “nnet.” Continuous variables are presented as mean ± standard deviation (SD) if normally distributed and were compared using the independent-sample *t*-test; otherwise, they were analyzed using the Mann–Whitney U test. Categorical variables were compared using the chi-square test. A two-tailed *p* value < 0.05 was considered statistically significant.

## 3 Results

### 3.1 Patients characteristics

These baseline characteristics are summarized in Table 1. Ultimately, a total of 744 patients with IAs were included in this study. The rates of ruptured IAs were 26.71% (113/423) in the training set and 27.72% (89/321) in the external validation set, with no statistically significant difference between the two sets (*p* = 0.823). Moreover, for the IAs ruptured group, the median time from symptom onset (or SAH onset) to blood sampling was 4.2 h [Inter quartile range (IQR): 2.5–7.8 h] in the training set and 3.8 h (IQR: 2.1–6.5 h) in the external validation set. The median time from hospital admission to CTA examination was 0.8 h (IQR: 0.15–2.3 h) in the training set and 1.2 h (IQR: 0.2–2.8 h) in the external validation set.

**Table 1:** The baseline characteristics of IAs patients.

Variables	Training set			External validation set		
	Rupture group (*N* = 113)	Unrupture group (*N* = 310)	*p* values	Rupture group (*N* = 89)	Unrupture group (*N* = 232)	*p* values
Gender			0.066			0.018
Female	70 (61.9%)	159 (51.3%)		69 (77.5%)	146 (62.9%)	
Male	43 (38.1%)	151 (48.7%)		20 (22.5%)	86 (37.1%)	
Age	61.1 (11.7)	66.6 (11.0)	<0.001	58.8 (11.8)	63.5 (12.1)	0.002
Position			<0.001			<0.001
Posterior communicating artery	16 (14.2%)	27 (8.7%)		36 (40.4%)	34 (14.7%)	
C7 segment of internal carotid artery	39 (34.5%)	190 (61.3%)		20 (22.5%)	134 (57.8%)	
Anterior communicating artery	47 (41.6%)	63 (20.3%)		15 (16.9%)	19 (8.1%)	
Middle cerebral artery	11 (9.7%)	30 (9.7%)		18 (20.2%)	45 (19.4%)	
Hypertension			0.239			0.919
No	28 (24.8%)	97 (31.3%)		40 (44.9%)	101 (43.5%)	
Yes	85 (75.2%)	213 (68.7%)		49 (55.1%)	131 (56.5%)	
Diabetes			0.199			0.054
No	92 (81.4%)	232 (74.8%)		81 (91.0%)	189 (81.5%)	
Yes	21 (18.6%)	78 (25.2%)		8 (9.0%)	43 (18.5%)	
Hyperlipidemia			0.278			0.005
No	93 (82.3%)	238 (76.8%)		57 (64.0%)	106 (45.7%)	
Yes	20 (17.7%)	72 (23.2%)		32 (36.0%)	126 (54.3%)	
Smoking			0.292			0.028
No	85 (75.2%)	215 (69.4%)		84 (94.4%)	196 (84.5%)	
Yes	28 (24.8%)	95 (30.6%)		5 (5.6%)	36 (15.5%)	
Drinking			0.198			0.056
No	89 (78.8%)	223 (71.9%)		85 (95.5%)	203 (87.5%)	
Yes	24 (21.2%)	87 (28.1%)		4 (4.5%)	29 (12.5%)	
WBC (x10^9^/L)	11.1 (4.17)	7.54 (2.69)	<0.001	12.6 (4.44)	6.86 (2.60)	<0.001
Neu (x10^9^/L)	9.15 (4.26)	5.36 (2.66)	<0.001	10.8 (4.29)	4.69 (2.42)	<0.001
Lym (x10^9^/L)	1.26 (0.81)	1.52 (0.86)	0.004	1.25 (0.81)	1.58 (0.64)	0.001
MO (x10^9^/L)	0.57 (0.27)	0.57 (0.47)	0.995	0.55 (0.55)	0.40 (0.17)	0.016
PLT (x10^9^/L)	207 (75.8)	217 (66.6)	0.225	224 (65.0)	212 (67.0)	0.142
Albumin (g/L)	41.2 (5.06)	41.1 (4.77)	0.968	42.3 (3.62)	42.1 (3.95)	0.734
Globulin (g/L)	29.2 (5.31)	29.4 (5.08)	0.739	29.7 (6.62)	29.9 (5.42)	0.868
ALT (U/L)	24.0 (26.3)	23.5 (16.2)	0.844	24.9 (24.4)	24.9 (22.4)	0.998
AST (U/L)	28.4 (17.8)	26.9 (15.9)	0.434	28.1 (25.9)	26.8 (19.3)	0.664
ALP (U/L)	89.2 (30.9)	91.6 (35.5)	0.491	78.4 (29.5)	86.8 (55.4)	0.081
GGT (U/L)	43.1 (96.5)	43.3 (68.2)	0.983	42.8 (64.6)	40.3 (46.2)	0.741
NLR	11.0 (8.68)	4.76 (4.41)	<0.001	12.6 (9.49)	3.59 (3.07)	<0.001
dNLR	1.71 (0.52)	1.51 (0.23)	0.021	1.53 (0.32)	1.40 (0.19)	0.001
PLR	225 (149)	179 (120)	0.003	241 (141)	154 (85.4)	<0.001
SII	2,283 (2104)	1,031 (1045)	<0.001	2,735 (2126)	759 (698)	<0.001
PNI	47.5 (6.83)	49.9 (18.4)	0.044	48.5 (5.86)	51.8 (26.9)	0.085
GLR	41.3 (81.5)	38.4 (74.1)	0.747	44.3 (66.5)	34.5 (79.9)	0.264
AGR	1.44 (0.27)	1.44 (0.28)	0.833	1.46 (0.23)	1.51 (0.98)	0.516
LMR	2.63 (1.82)	3.25 (1.72)	0.002	2.97 (1.92)	4.32 (1.93)	<0.001
APRI	0.17 (0.16)	0.14 (0.11)	0.103	0.15 (0.22)	0.17 (0.27)	0.645
AAR	1.42 (0.62)	1.33 (0.63)	0.200	1.33 (0.59)	1.23 (0.62)	0.174
ALRI	31.7 (29.9)	23.4 (26.5)	0.010	30.3 (28.6)	20.6 (21.1)	0.004
ANRI	4.67 (10.7)	6.28 (5.17)	0.127	3.11 (3.18)	7.16 (8.87)	<0.001
BPRI	0.13 (0.15)	0.12 (0.10)	0.384	0.16 (0.33)	0.15 (0.27)	0.797
SIRI	6.36 (6.77)	2.73 (3.74)	<0.001	5.92 (5.26)	1.49 (1.68)	<0.001
NγLR	418 (1204)	208 (389)	0.071	526 (964)	176 (486)	0.001
GPR	0.26 (0.91)	0.22 (0.32)	0.583	0.21 (0.29)	0.28 (0.79)	0.249

WBC, white blood cell count; Neu, neutrophil count; Lym, lymphocyte count; MO, monocyte count; PLT, platelet count; ALT, alanine aminotransferase; AST, aspartate aminotransferase; ALP, alkaline phosphatase; GGT, Gamma-glutamyl transferase; NLR, Neutrophil-to-lymphocyte ratio; dNLR, (White blood cell-neutrophil)-to-lymphocyte ratio; PLR, Platelet-to-lymphocyte ratio; SII, Systemic immune-inflammation index; PNI, Albumin+5 * lymphocytes; GLR, Gamma-glutamyl transpeptidase-to-lymphocyte ratio; AGR, Albumin-to-globulin ratio; LMR, Lymphocyte-to-monocyte ratio; APRI, Aminotransferase-to-platelet ratio; AAR, Aminotransferase-to-alanine aminotransferase ratio; ALRI, Aminotransferase-to-lymphocyte ratio; ANRI, Aminotransferase-to-neutrophil ratio; BPRI, Alanine aminotransferase-platelet ratio; SIRI, Systemic inflammation response index; GPR, Gamma-glutamyl transferase-to-platelet ratio; NγLR, Neutrophil times gamma-glutamyl transpeptidase to lymphocyte ratio; IAs, Intracranial aneurysm.

### 3.2 Clinical and radiomics feature selection

For 27 serum biomarkers, the LASSO regression and ten fold cross validation (Supplementary Figure 2) were used to screen the features, with 6 most important features selected. Ultimately, the SIS for differentiating ruptured from unruptured IAs is as follows:

$$\begin{array}{l} \text{SIS}=-2.757+0.126^{*}\text{WBC}+0.102\\ {*}^{\text{Neu}}-0.071^{*}\text{MO}-0.002^{*}\text{PLT}+\\ 0.044^{*}\text{NLR}+0.631^{*}\text{APRI} \end{array}$$

For radiomics, the radiomic features with poor reproducibility and ICC < 0.80 were eliminated. After a series of feature dimensionality reduction, the mRMR was gradually used to screen for the features with the highest redundancy. The number of feature filtering process is summarized in Supplementary Table 4. Furthermore, Figure 2 summarizes the detailed radiomics features screening of Intra-aneurysm, Peri-aneurysmal 1 mm, and Intra-perianeurysmal 1 mm based on SHAP algorithm and LASSO regression. Ultimately, the CRS was calculated based on the IRSIAs, PRSIAs, and IPRSIAs. Supplementary Table 5 provides a detailed calculation processes of the IRSIAs, PRSIAs, IPRSIAs, and CRS.

**Figure 2:**
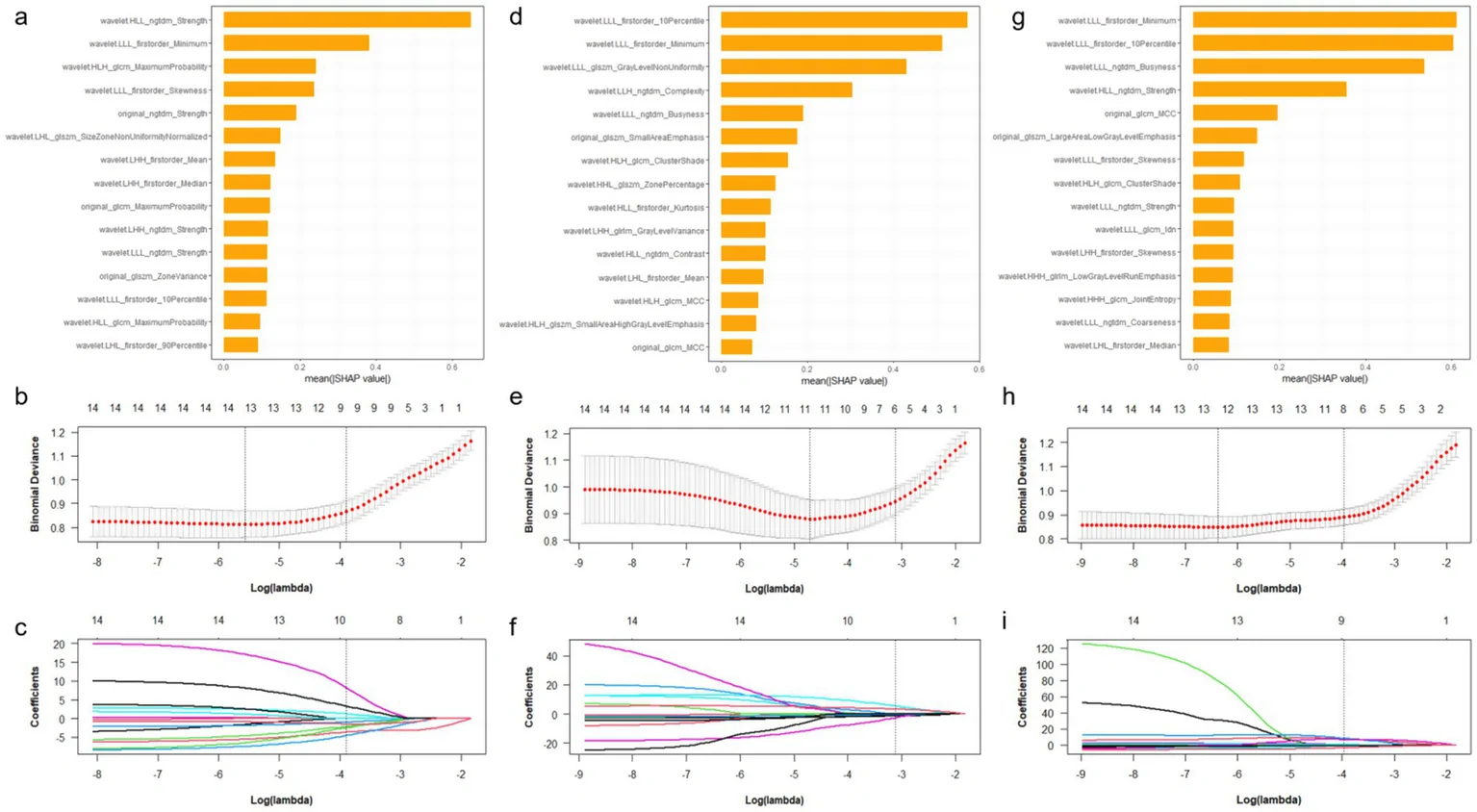
Radiomics features screening using SHAP algorithm and LASSO regression. The top fifteen most contributive radiomics features and the final modeling features are retained through the SHAP algorithm **(a,d,g)**, tenfold cross-validation **(b,e,h)** and LASSO regression **(c,f,i)** for Intra-aneurysm **(a–c)**, Peri-aneurysmal 1 mm **(d–f)**, and Intra-perianeurysmal 1 mm **(g–i)**. SHAP, the SHapley Additive exPlanations algorithm; LASSO, least absolute shrinkage and selection operator.

The performance of IRSIAs, PRSIAs, IPRSIAs, and CRS for differentiating ruptured from unruptured IAs is summarized in Table 2. Supplementary Figures 3a,b suggests that the peri-aneurysmal 1 mm radiomics has supplementary value for intra-aneurysm for differentiating ruptured from unruptured IAs in training set (AUC: increased from 0.870 to 0.888) and external validation set (AUC: increased from 0.781 to 0.814), and there was a statistically significant difference in training set (DeLong test: *Z* = 2.423, *p* = 0.0154) and external validation set (DeLong test: *Z* = 2.081, *p* = 0.0375).

**Table 2:** The performance of radiomics features within and around the IAs for predicting IAs rupture in training set and external validation set.

Features	Training set			External validation set		
	AUC	SE	95%CI	AUC	SE	95%CI
IRSIAs	0.870	0.0194	0.834 to 0.900	0.781	0.0309	0.732 to 0.825
PRSIAs	0.845	0.0208	0.807 to 0.878	0.765	0.0288	0.715 to 0.811
IPRSIAs	0.854	0.0200	0.817 to 0.887	0.819	0.0243	0.773 to 0.860
CRS	0.891	0.0175	0.857 to 0.919	0.828	0.0282	0.782 to 0.868

AUC, area under the curve; CI, confidence interval; SE, standard error; IRSIAs, the radiomics score based on intra-aneurysm of IAs; PRSIAs, the radiomics score based on peri-aneurysmal 1 mm of IAs; IPRSIAs, the radiomics score based on intra-perianeurysmal 1 mm of IAs; CRS, the combined radiomics score based on intra-aneurysm, perianeurysmal 1 mm and intra-perianeurysmal 1 mm of IAs; IAs, Intracranial aneurysm.

### 3.3 Independent predictors for IAs rupture

In the training set, the univariate and multivariate logistic regression analysis were used for screening features, and it was summarized in Table 3. Eventually, the results indicated that CRS [OR = 2.467 (95%CI 1.968–3.092); *p* < 0.001], and SIS [OR = 2.852 (95%CI 2.032–4.002); *p* < 0.001] were independent predictors for differentiating ruptured from unruptured IAs.

**Table 3:** The univariate and multivariate logistic regression analysis for screening features in training set.

Variables	Univariate analysis			Multivariate analysis		
	OR	95%CI	*p* values	OR	95%CI	*p* values
CRS	2.718	2.200–3.359	<0.001	2.467	1.968–3.092	<0.001
Gender	0.655	0.422–1.018	0.060			
Age	0.959	0.941–0.978	<0.001	0.975	0.949–1.003	0.081
Position of IAs						
Posterior communicating artery	Reference					
C7 segment of internal carotid artery	0.491	0.224–1.080	0.077			
Anterior communicating artery	0.794	0.385–1.639	0.533			
Middle cerebral artery	0.475	0.165–1.045	0.087			
Hypertension (Yes *vs*. No)	0.723	0.443–1.181	0.195			
Diabetes (Yes *vs*. No)	1.473	0.859–2.525	0.159			
Hyperlipidemia (Yes *vs*. No)	1.407	0.811–2.439	0.224			
Smoking (Yes *vs*. No)	1.341	0.821–2.191	0.241			
Drinking (Yes *vs*. No)	1.447	0.865–2.420	0.159			
SIS	3.518	2.653–4.667	<0.001	2.852	2.032–4.002	<0.001

OR, odds ratio; CI, confidence interval; IAs, intracranial aneurysm; SIS, the serum inflammation score was constructed using logistic regression; CRS, the combined radiomics score based on intra-aneurysm, perianeurysmal 1 mm and intra-perianeurysmal 1 mm of IAs.

Supplementary Figures 3c,d suggests that the SIS has supplementary value for CRS for differentiating ruptured from unruptured IAs in training set (AUC: increased from 0.891 to 0.923) and external validation set (AUC: increased from 0.828 to 0.873), and there was a statistically significant difference in training set (DeLong test: *Z* = 2.984, *p* = 0.0028) and external validation set (DeLong test: *Z* = 3.152, *p* = 0.0016).

### 3.4 Model construction and model fusion

Seven machine learning models of Bayes, DecisionTree, KNN, LRLASSO, NNet, SVM, and XGBoost were established to evaluate performance for differentiating ruptured from unruptured IAs based on the CRS and SIS. Supplementary Figure 4 summarizes ROC curves and confusion matrices of seven machine learning.

Moreover, based on the prediction probabilities of all machine learning models, several fusion strategies of the aMSLM, gMSLM, wMSLM, and LRSLM were integrated by using arithmetic mean, geometric mean, weighted mean, and logistic regression. Figure 3 summarizes confusion matrices of four fusion models. Importantly, LRSLM demonstrated superior predictive performance compared to the aMSLM, gMSLM, and wMSLM in training set (AUC:0.9880 *vs.* 0.9516 *vs.* 0.9444 *vs.* 0.9589; DeLong test, all *p* < 0.0001), and there was no statistically significant difference in the external validation set (AUC:0.9177 *vs.* 0.8873 *vs.* 0.8839 *vs.* 0.8889; DeLong test, *p* = 0.0793, 0.0658, 0.0873). The 11 models for differentiating ruptured from unruptured IAs are summarized in Table 4 and Supplementary Table 6.

**Figure 3:**
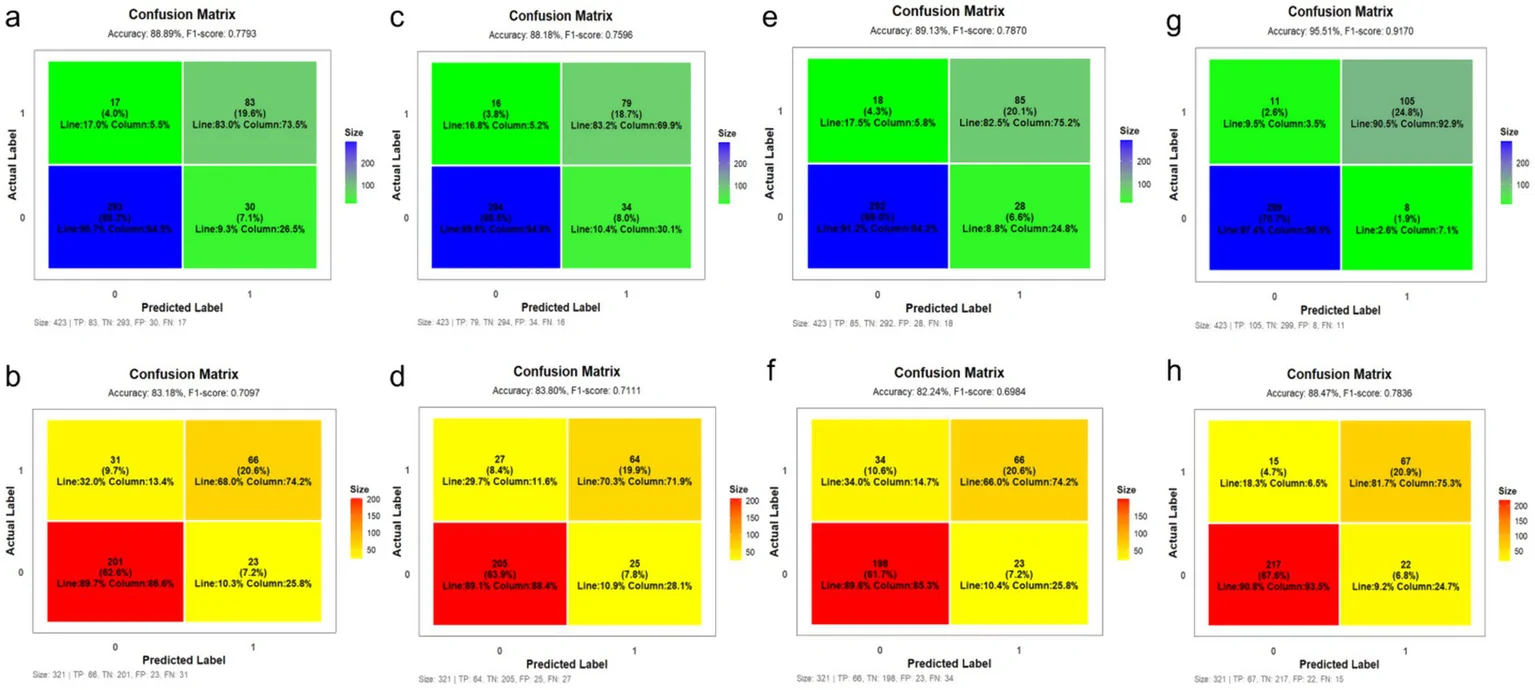
Confusion matrix of aMSLM **(a,b)**, gMSLM **(c,d)**, wMSLM **(e,f)**, LRSLM **(g,h)** for predicting IAs rupture in training set **(a,c,e,g)** and external validation set **(b,d,f,h)**. aMSLM, the ensemble model was integrated for all machine learning prediction probability using arithmetic mean; gMSLM, the ensemble model was integrated for all machine learning prediction probability using geometric mean; wMSLM, the ensemble model was integrated for all machine learning prediction probability using weighted mean; LRSLM, the ensemble model was integrated for all machine learning prediction probability using logistic regression; IAs, Intracranial aneurysm.

**Table 4:** The perfomance of 11 models for differentiating ruptured from unruptured IAs in external validation set.

Models	AUC (95%CI)	Accuracy	Precision	Recall	F1_Score	Brier_Score
Bayes	0.8329 (0.7824–0.8834)	0.8131	0.6854	0.6559	0.6703	0.1579
DecisionTree	0.8545 (0.8022–0.9067)	0.8692	0.7528	0.7701	0.7614	0.1169
KNN	0.8863 (0.8447–0.9278)	0.8162	0.7191	0.6531	0.6845	0.1178
LRLASSO	0.8724 (0.8251–0.9197)	0.8318	0.6923	0.7079	0.7000	0.1200
NNet	0.8848 (0.8403–0.9293)	0.7975	0.7416	0.6111	0.6701	0.1209
SVM	0.8509 (0.7943–0.9074)	0.8287	0.6966	0.6889	0.6927	0.1230
XGBoost	0.8848 (0.8429–0.9267)	0.8100	0.6228	0.7978	0.6995	0.1361
aMSLM	0.8873 (0.8447–0.9298)	0.8318	0.7416	0.6804	0.7097	0.1165
gMSLM	0.8839 (0.8391–0.9287)	0.8308	0.7191	0.7033	0.7111	0.1141
wMSLM	0.8889 (0.8471–0.9308)	0.8224	0.7416	0.6600	0.6984	0.1177
LRSLM	0.9177 (0.8855–0.9499)	0.8847	0.7528	0.8171	0.7836	0.0999

KNN, k-nearest neighbor; LRLASSO, The logistic regression based on least absolute shrinkage and selection operator; NNet, neural network; SVM, support vector machine; XGBoost, extreme gradient boosting classifiers; DecisionTree, Decision Tree; aMSLM, the ensemble model was integrated for all machine learning prediction probability using arithmetic mean; gMSLM, the ensemble model was integrated for all machine learning prediction probability using geometric mean; wMSLM, the ensemble model was integrated for all machine learning prediction probability using weighted mean; LRSLM, the ensemble model was integrated for all machine learning prediction probability using logistic regression; AUC, area under the curve; CI, Confidence interval; IAs, Intracranial aneurysm.

### 3.5 Model evaluation and model validation

In the external validation set, the evaluation and validation of the LRSLM are summarized in Figure 4. When the cutoff was 0.5, the LRSLM achieved an accuracy of 0.8847, Precision of 0.7528, Recall of 0.8171, F1 score of 0.7836 and Brier score of 0.0999. The DCA suggested that LRSLM achieved more clinical net benefits with a threshold probability ranging from 10 to 85%. The calibration curve suggested that LRSLM owned good consistency with a Brier score of 0.0999 in external validation set, and the Hosmer–Lemeshow goodness-of-fit test suggested that there was no significant difference between predicted and observed values and confirming good model calibration in external validation set (*χ*^2^ = 6.7116, *p* = 0.568). Moreover, the CIC indicated that LRSLM could also yield greater net benefits across a wide threshold range, suggesting that the LRSLM might be worthy of being promoted for use in clinical practice.

**Figure 4:**
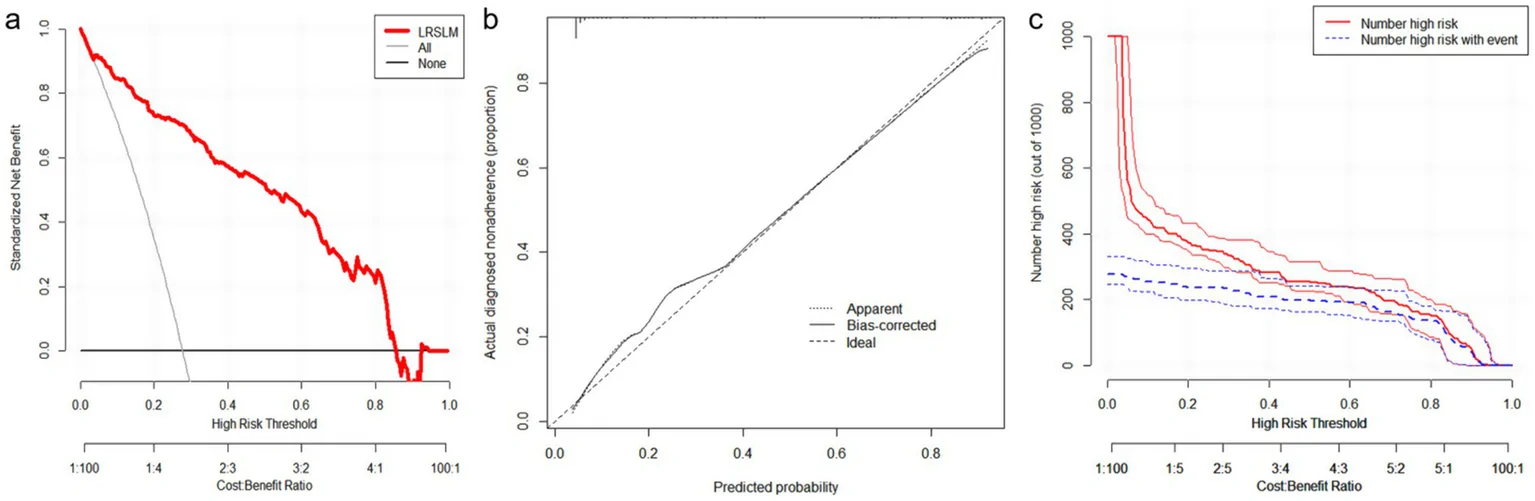
Model verification and evaluation of the LRSLM. The DCA curve **(a)**, calibration curve **(b)** and clinical impact curve **(c)** of LRSLM for differentiating ruptured from unruptured IAs in external validation set. LRSLM, the ensemble model was integrated for all machine learning prediction probability using logistic regression; DCA, clinical decision curve analysis; IAs, Intracranial aneurysm.

Moreover, a total of 1,000 Bootstrap samples (with replacement) were drawn from the training set, with each subjected to the full feature selection workflow employed in the original pipeline. For the LRSLM, this 1,000 Bootstrap Resampling yielded an AUC of 0.918 (95% CI: 0.884–0.946) on the external validation set (Supplementary Figure 5). In addition, given the inherent class imbalance in our dataset (approximately 27% rupture prevalence), we prioritized balanced accuracy and precision-recall AUC (PR-AUC) alongside ROC-AUC. The LRSLM achieved a balanced accuracy of 0.8605 and a PR-AUC of 0.887 in the external validation set, confirming robust performance across both classes and supporting the clinical relevance of our model.

Due to the different locations of IAs within the brain, we conducted subgroup analyses of different positions (Posterior communicating artery *vs*. C7 segment of internal carotid artery *vs*. Anterior communicating artery *vs*. Middle cerebral artery) to explore the discriminatory efficacy of LRSLM in external validation set. The results showed that the LRSLM model maintained good discriminative performance at four different subgroup positions (AUC range: 0.847 to 0.903; Supplementary Table 7), and there was no statistically significant difference between different positions (DeLong test, all *p* > 0.05). This indicates that the model also has good generalization ability in different anatomical positions.

### 3.6 Model interpretability analysis

As shown in Figures 5a–c, the intra-aneurysmal, peri-aneurysmal 1 mm, and intra-perianeurysmal 1 mm radiomics features have a strong positive correlation (Spearman’s r: 0.62, 0.72, 0.84; all *p* < 0.001), indicating that the peri-aneurysmal 1 mm and intra-aneurysmal radiomics features capture common pathological microenvironmental changes and achieving a synergistic effect in quantifying the rupture of IAs. Importantly, correlation analysis (Figures 5d–g) also demonstrated a good positive correlation (Spearman’s r: 0.32, 0.29, 0.32, 0.34; all *p* < 0.001) between the IRSIAs, PRSIAs, IPRSIAs, CRS and SIS. This finding may suggest that peri-aneurysmal 1 mm and intra-aneurysmal radiomics features and serum inflammatory biomarkers—potentially reflecting pathological microenvironment interactions of IAs and the average value of systemic serum inflammatory burden—played a dominant role in enhancing the predictive accuracy of the fusion model.

**Figure 5:**
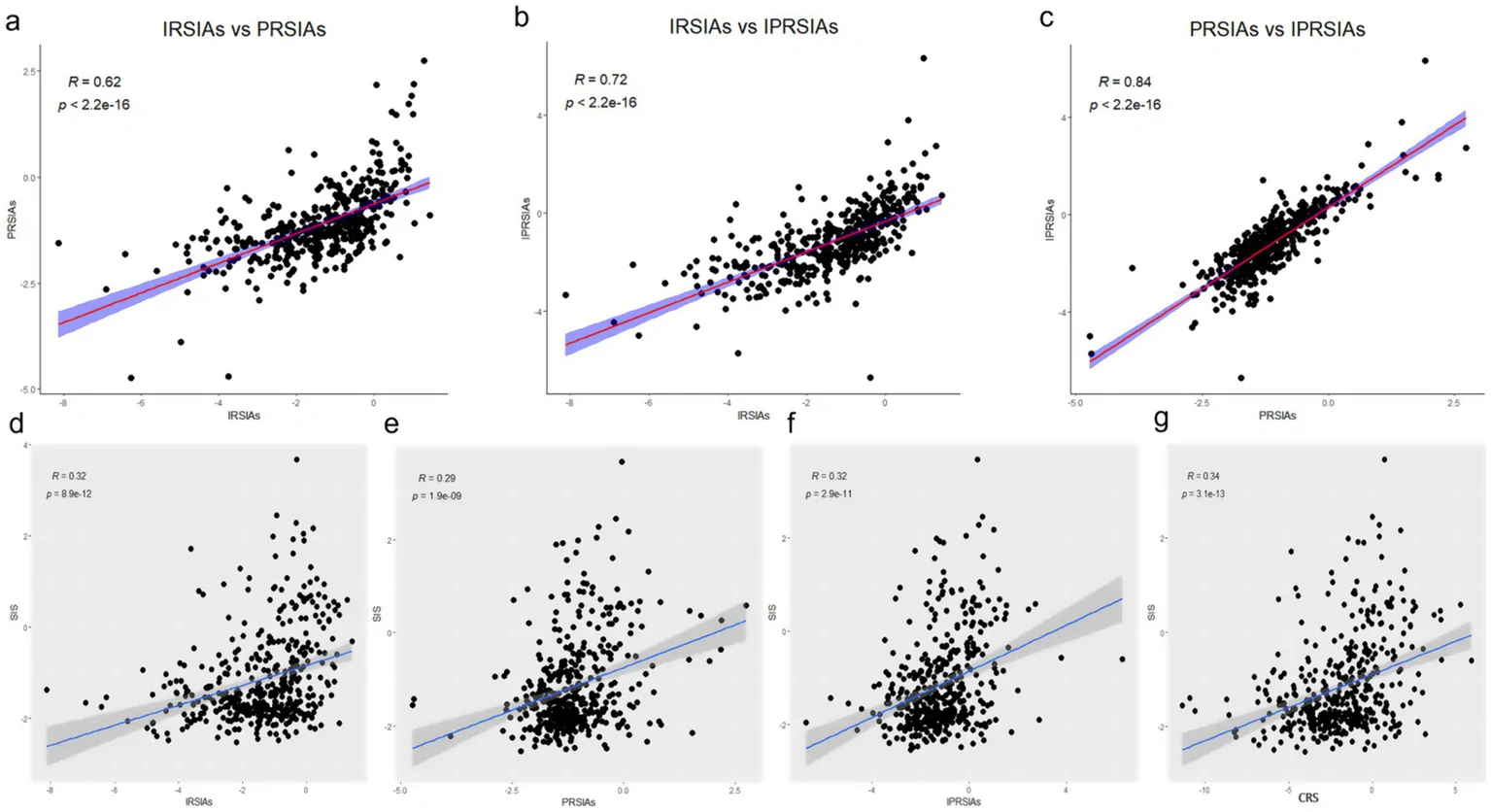
Correlation analysis. The scatter plot suggests a significant positive correlation for IRSIAs and PRSIAs **(a)**, IRSIAs and IPRSIAs **(b)**, PRSIAs and IPRSIAs **(c)**. Correlation analysis suggests a good positive correlation for the IRSIAs and SIS **(d)**, PRSIAs and SIS **(e)**, IPRSIAs and SIS **(f)**, CRS and SIS **(g)**. IRSIAs, the radiomics score based on intra-aneurysm of IAs; PRSIAs, the radiomics score based on perianeurysmal 1 mm of IAs; IPRSIAs, the radiomics score based on intra-perianeurysmal 1 mm of IAs; CRS, the combined radiomics score based on intra-aneurysm, perianeurysmal 1 mm and intra-perianeurysmal 1 mm of IAs; SIS, the serum inflammation score was constructed using logistic regression; IAs, Intracranial aneurysm.

RCS analysis showed a significant nonlinear correlation (Nonlinear *p* < 0.05; Overall *p* < 0.05) between IRSIAs, PRSIAs and the increased incidence of IAs rupture; However, no nonlinear relationship was observed between IPRSIAs and SIS. As shown in Figure 6, when these features are at a lower level, they are associated with a decrease in the incidence of IAs rupture; However, as the values of these indicators increase, the ratio gradually increases.

**Figure 6:**
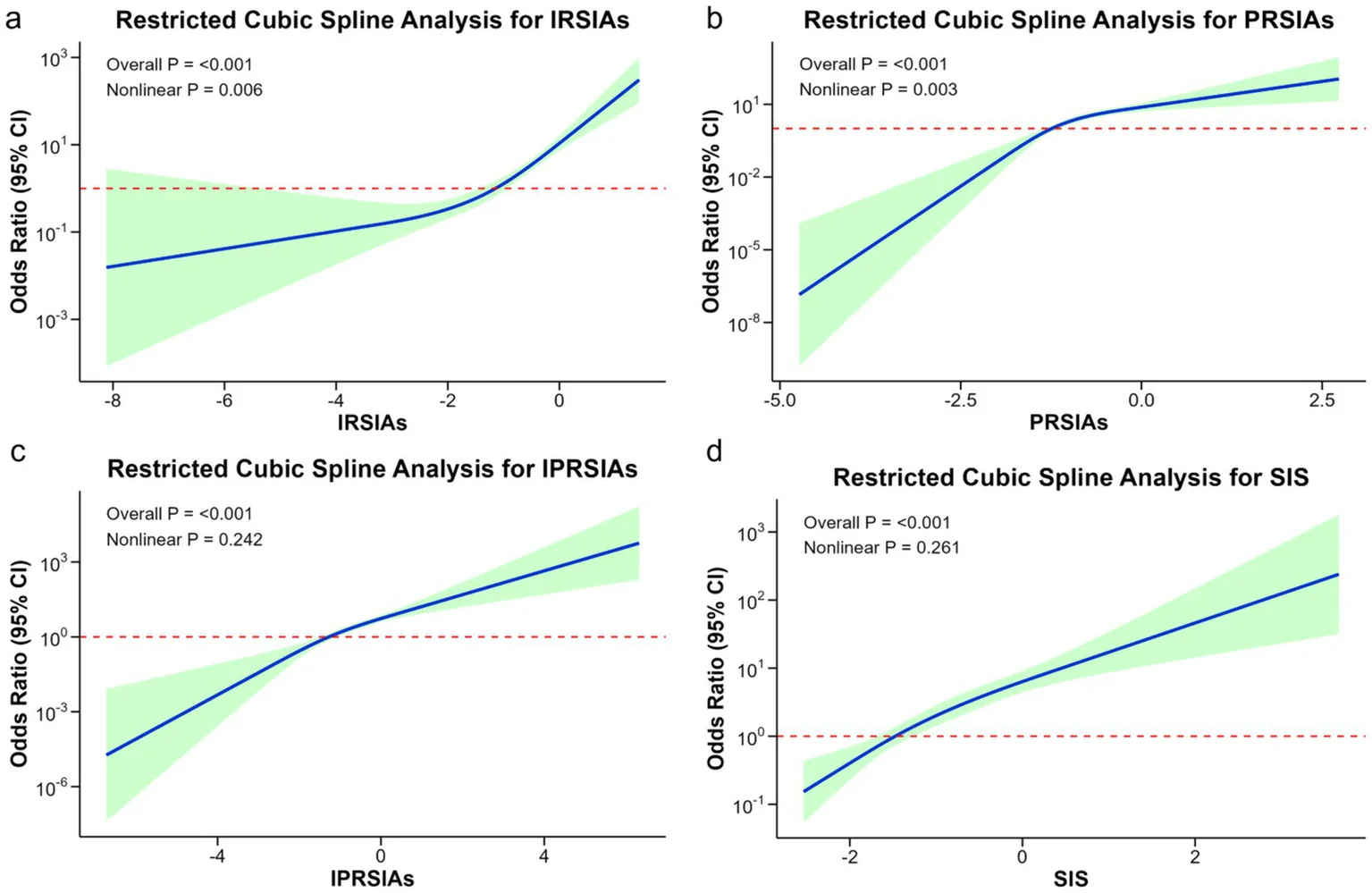
Restrictive cubic spline analysis for IRSIAs **(a)**, PRSIAs **(b)**, IPRSIAs **(c)**, and SIS **(d)**. RCS analysis showed a significant nonlinear correlation (Nonlinear *p* < 0.05; Overall *p* < 0.05) between IRSIAs, PRSIAs and the increased incidence of IAs rupture; However, no nonlinear relationship was observed between IPRSIAs and SIS. IRSIAs, the radiomics score based on intra-aneurysm of IAs; PRSIAs, the radiomics score based on perianeurysmal 1 mm of IAs; IPRSIAs, the radiomics score based on intra-perianeurysmal 1 mm of IAs; SIS, the serum inflammation score was constructed using logistic regression; IAs, Intracranial aneurysm.

SHAP can enhance the interpretation and visualization of the importance of each feature in the optimal model. As shown in Figures 7a,b, there were 23 radiomics features from Intra-aneurysm, Peri-aneurysmal 1 mm, and Intra-perianeurysmal 1 mm, and the Peri_wavelet.LLL_firstorder_10Percentile contributed the most to the model, achieving a Shapley value mean of 0.0893. Notably, among the 7 machine learning models, the XGBoost made the greatest contribution, with a Shapley value mean of 0.3777 (Figure 7c). Furthermore, the Intra-aneurysmal radiomics score contributed the most to the final fusion model, followed by SIS (Figure 7d).

**Figure 7:**
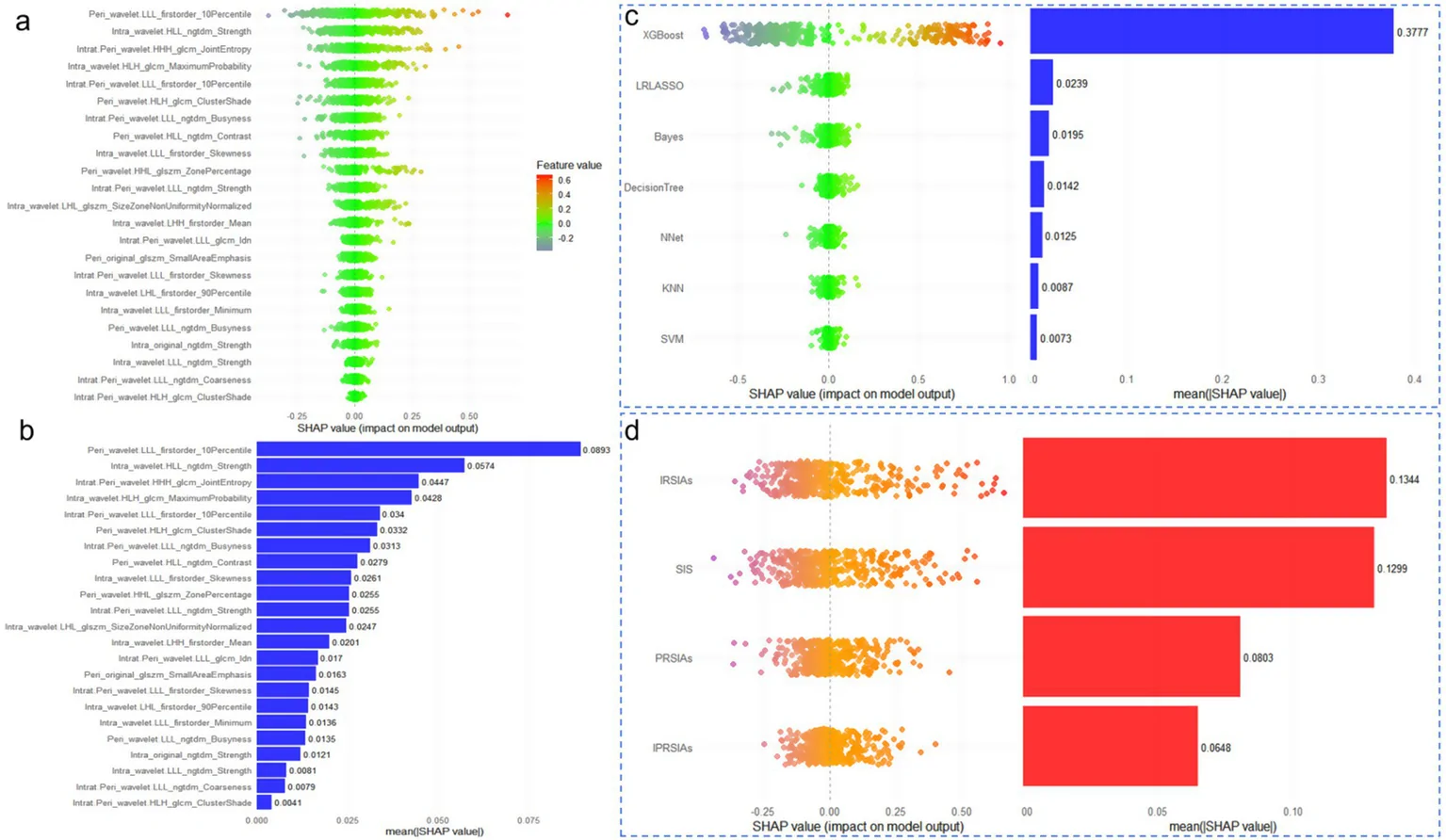
SHAP analysis for model interpretability. **(a)** SHAP summary plot for the 23 radiomics features, and the top three radiomics features were Peri_wavelet. LLL_firstorder_10Percentile, Intra_wavelet. HLL_ngtdm_Strength, and Intra-Peri_wavelet. HHH_glcm_JointEntropy, with average Shapley values of 0.0893, 0.0574, and 0.0447 **(b)**. **(c)** SHAP summary plot for the 7 machine learning, and the XGBoost achieved the greatest contribution with an average Shapley value of 0.3777. SHAP analysis also showed that the IRSIAs contributed the most to the final fusion LRSLM model, followed by SIS **(d)**. SHAP, the SHapley Additive exPlanations algorithm; IRSIAs, the radiomics score based on intra-aneurysm of IAs; PRSIAs, the radiomics score based on perianeurysmal 1 mm of IAs; IPRSIAs, the radiomics score based on intra-perianeurysmal 1 mm of IAs; StackingRS, the stacking radiomics score based on intra-aneurysm, perianeurysmal 1 mm and intra-perianeurysmal 1 mm of IAs; SIS, the serum inflammation score was constructed using logistic regression; IAs, Intracranial aneurysm.

## 4 Discussion

From a biological perspective, serum inflammatory biomarkers reflect the average systemic inflammatory burden, while CTA based intra-aneurysmal and peri-aneurysmal 1 mm radiomics features characterize changes in blood flow dynamics, aneurysm wall, and perianeurysmal ecological microenvironment of IAs. This study innovatively found that serum inflammatory biomarkers and perianeurysmal 1 mm radiomics features can enhance the diagnostic efficacy of CTA for differentiating ruptured from unruptured IAs. Importantly, this study constructed and externally validated an interpretable model LRSLM, which performed well in the external validation set (AUC of 0.9177) with good calibration, and achieved clinical net benefits at a wide threshold of 10–85%. This finding may suggest that intra-aneurysmal and peri-aneurysmal 1 mm radiomics features and serum inflammatory biomarkers—potentially reflecting pathological microenvironment interactions of IAs and the average value of systemic serum inflammatory burden—played a dominant role in enhancing the predictive accuracy of the fusion model, which may help optimize clinical intervention decisions for high-risk ruptured IAs.

The SIS constructed in this study integrates serum inflammatory biomarkers such as white blood cells, neutrophils, monocytes, platelets, NLR, and APRI. Previous studies have shown that the inflammatory response of the aneurysm wall is a key factor leading to the destruction of its structural integrity, matrix degradation, and eventual rupture (13–18, 23). Among them, NLR, as a biomarker of systemic inflammation and immune imbalance, is closely related to neutrophil infiltration and lymphocyte immune regulation imbalance in the aneurysm wall (17, 18). However, we must point out that SIS reflects the average level of systemic inflammatory load, and the blood samples in the ruptured group were obtained shortly after ictus, during the hyper-acute phase of SAH. During this period, massive sympathetic activation, release of damage-associated molecular patterns, and breakdown of the blood–brain barrier trigger a robust systemic inflammatory cascade, manifesting as elevated neutrophil counts and NLR. These changes are well-documented physiological responses to acute intracranial hemorrhage and are therefore not specific to pre-existing aneurysm wall vulnerability. Consequently, the contribution of the SIS may be interpreted as capturing the host’s acute reaction to hemorrhage, rather than a pre-morbid risk factor. More specifically, the serum indicators capture the host’s real-time physiological status at presentation, which may be useful for acute phenotypic characterization and for contextualizing the CTA radiomic signals.

Notably, the radiomics features of the tumor can non invasively quantify the microscopic heterogeneity around the tumor, reflecting pathological changes such as the tumor microenvironment, cell composition, inflammatory infiltration and matrix remodeling, and it has been widely used in the field of tumor research (28–30). However, the occurrence and rupture of IAs are not limited to the aneurysm wall itself, and the surrounding microenvironment also plays an important role (31–34). This study innovatively incorporated radiomics features within a 1 mm radius around the aneurysm, aiming to capture the interaction between the aneurysm cavity, aneurysm wall, and surrounding tissues such as subarachnoid space, brain parenchyma, and perivascular spaces. Notably, the results showed a significant positive correlation between radiomics features within the intra-aneurysm and peri-aneurysmal 1 mm (Spearman’s r: 0.62, 0.72, and 0.84, respectively), supporting the idea that these three regions have a synergistic promoting effect in the predictive model. This effectively revealed that the 1 mm area around the aneurysm may reflect pathological changes such as cell composition, inflammatory infiltration, and matrix remodeling of the IAs wall, as well as key inflammatory microenvironment information including the outward spread of aneurysm wall inflammation, peripheral tissue reactions caused by local hemodynamic changes, and dysfunction of perivascular adipose tissue (32, 33). Moreover, adipose tissue in the 1 mm microenvironment around the aneurysm can secrete various pro-inflammatory cytokines, further exacerbating the inflammatory response and oxidative stress of the aneurysm wall (35, 36). Therefore, the synergistic effect of peritumoral radiomics features may stem from its capture of dynamic interactions between the aneurysm and the surrounding microenvironment, providing supplementary information for a more comprehensive and accurate assessment of the biological instability of aneurysm rupture.

Importantly, SHAP analysis further revealed the intrinsic logic of model decision-making, with radomics feature of Peri_wavelet.LLL_firstorder_10Percentile contributing the most to the model (average Shapley value of 0.0893). This feature originates from the 1 mm area around the IAs and belongs to the first-order statistic of the low-pass filtering sub-band after wavelet transform. It reflects the lower percentile of the pixel grayscale distribution in this area, which may correspond to low-density or low metabolic regions in the aneurysm wall or surrounding tissue of the IAs. The prominent contribution of this feature may suggest that subtle heterogeneity in local tissue density or metabolism in the aneurysm wall or surrounding microenvironment may be a key imaging phenotype reflecting the unstable state of aneurysms. Moreover, this feature likely captures variations in local contrast distribution, which may be influenced by peri-aneurysmal flow dynamics, partial volume effects, or subtle peri-aneurysmal tissue changes (e.g., edema or micro-hemorrhage). It is worth noting that in the final fusion model, the XGBoost algorithm made the most significant contribution (with an average Shapley value of 0.3777), which may be attributed to its ability to effectively capture complex nonlinear interactions between multiple features through gradient boosting strategy, especially suitable for integrating heterogeneous data such as radiomics and serum biomarkers. Importantly, the correlation analyses revealed strong positive associations between intra-aneurysmal and peri-aneurysmal radiomic features, as well as significant positive correlations between the intra−/perianeurysmal radiomics and SIS (all *p* < 0.001). RCS analysis further demonstrated a significant non-linear relationship between these radiomic features and an increased incidence of IAs rupture. This discovery may indicate that radiomics features surrounding and within IAs, as well as serum inflammatory biomarkers—which may reflect the interaction between the inflammatory microenvironment and the average systemic serum inflammatory load—play a dominant role in enhancing the predictive accuracy of fusion models. Additionally, SIS, as a quantitative indicator of serum systemic inflammation, reflects the overall immune status of the body and provides a supplementary perspective from “local” to “systemic” for CTA radiomics in differentiating ruptured from unruptured IAs. Moreover, multiple machine learning algorithms can integrate the advantages of each machine learning through logical regression stacking strategy fusion, effectively avoid over fitting or deviation of a single model, and thus build a fusion model with stronger generalization ability. Therefore, this model integrates multiple machine learning algorithms, CTA radiomics of intra−/perianeurysmal microenvironments, and systemic inflammatory metabolic status of the body, combined with advanced fusion strategies, to achieve multidimensional and high-precision assessment of the risk of IAs rupture. This enables a more comprehensive evaluation of the biological behavior of IAs rupture from the perspectives of systemic metabolism and immune environment.

However, given the known sensitivity of radiomic features to acquisition parameters, scanner heterogeneity is an important consideration. Although all CTA images were resampled to 1-mm isotropic voxels, intensity-normalized, and discretized with a fixed bin width, residual scanner-related variability may persist. We considered ComBat harmonization; however, because ComBat requires predefined batch labels and may remove biologically meaningful variance, particularly in small batches, it was not applied as the primary approach. Instead, the external validation cohort was acquired using different scanners and protocols, providing a stringent test of generalizability. The small uCT780 subgroup may limit definitive conclusions. Prospective multicenter studies with balanced scanner distributions and harmonization methods such as ComBat are warranted to further validate and refine the model.

Additionally, this study also has certain limitations. Firstly, this study is retrospective and some SAH patients were excluded without undergoing CTA examination. The training and external validation sets were collected over non-overlapping time periods, which may introduce temporal biases due to potential changes in clinical practice, patient selection, or unmeasured confounders. Secondly, for patients with partially ruptured IAs, it is difficult to obtain CTA images before rupture. Additionally, based on CTA, the 1 mm area around IAs not only characterizes the aneurysm wall, but also cannot fully reflect the interaction between the IAs wall and the surrounding inflammatory microenvironment stimuli. Perhaps the wider area around IAs also has supplementary value in quantifying IAs rupture. Furthermore, the hemodynamic parameters of quantified IAs were not analysed in the study, nor were they included in the morphological parameters of IAs. Computational fluid dynamics (CFD) simulations can quantify parameters such as wall shear stress (WSS), oscillatory shear index (OSI), etc., which are important supplements for assessing the biomechanical state and rupture risk of aneurysms. Thirdly, this study only included single IAs to avoid interdependence within patients and ensure precise segmentation of the area surrounding the aneurysm. Furthermore, the vascular spasm and other SAH-related complications were not included as exclusion criteria. Since these complications themselves can trigger significant systemic inflammatory responses. Therefore, prospective design is needed to further validate the effectiveness of this model in multiple aneurysms. Finally, although the model has been validated through external validation, there is still a lack of prospective cohort data from multiple centers and regions for further validation, which will be the direction of our future efforts.

## 5 Conclusion

This LRSLM model demonstrates robust and well-calibrated performance in discriminating ruptured from unruptured IAs in an external validation set. It provides a supplementary perspective from “local” to “systemic” for CTA radiomics in differentiating ruptured from unruptured IAs, and further comprehensively evaluates the biological behavior of IAs rupture from the perspective of systemic metabolism and immune environment. The Peri_wavelet.LLL_firstorder_10Percentile radiomic feature and the XGBoost base classifier contributed most substantially to the fusion model. SHAP-based interpretability analysis enhances the clinical acceptability of the model by providing transparent decision-level explanations. However, given the cross-sectional design, this findings may be interpreted as hypothesis-generating evidence for rupture-associated phenotypes. Prospective longitudinal studies are urgently needed to determine whether these features can predict future rupture events, which would represent the true translational goal.

## Data Availability

The original contributions presented in the study are included in the article/Supplementary material, further inquiries can be directed to the corresponding authors.
