# Supplementary material for: Interpretable model using serum biomarkers and intra-/peri-aneurysmal CTA radiomics for discriminating ruptured from unruptured intracranial aneurysms: an external validation retrospective study
Source: Front Neurol. 2026 Oct 7;17:1867021. doi: 10.3389/fneur.2026.1867021 (PMC13683125; doi:10.3389/fneur.2026.1867021)
Supplement: Supplementary file 1 [file Data_Sheet_1.PDF]

Supplementary Material

1.Supplementary Tables

Supplementary Table 1：

The definition formulas for 16 inflammatory-related indicators.

Definition formula of serum biomarkers	Calculation formula	Abbreviations
Neutrophil-to-lymphocyte ratio	Neu/Lym	**NLR**
(White blood cell-neutrophil)-to-lymphocyte ratio	(WBC-Neu)/Lym	**dNLR**
Platelet-to-lymphocyte ratio	PLT/Lym	**PLR**
Systemic immune-inflammation index	Neu*PLT/Lym	**SII**
Albumin+5 * lymphocytes	ALB+5 * Lym	**PNI**
Gamma-glutamyl transpeptidase-to-lymphocyte ratio	GGT/Lym	**GLR**
Albumin-to-globulin ratio	ALB/GLOB	**AGR**
Alanine aminotransferase-platelet ratio	ALT/PLT	**BPRI**
Lymphocyte-to-monocyte ratio	Lym/MO	**LMR**
Aminotransferase-to-platelet ratio	AST/PLT	**APRI**
Aminotransferase-to-alanine aminotransferase ratio	AST/ALT	**AAR**
Aminotransferase-to-lymphocyte ratio	AST/Lym	**ALRI**
Aminotransferase-to-neutrophil ratio	AST/Neu	**ANRI**
Systemic inflammation response index	(Neu*MO)/Lym	**SIRI**
Gamma-glutamyl transferase-to-platelet ratio	GGT/PLT	**GPR**
Neutrophil times gamma-glutamyl transpeptidase to lymphocyte ratio	GGT*Neu/Lym	**NγLR**

Supplementary Table 2：

All IAs patient underwent CTA scanning using Revlution 256 spiral CT (N=423; *General GE, USA*), 256 spiral iCT (N=280; *General Philips, Netherlands*) and uCT 780 (N=41; *General GE, China*). A 1.2 – 1.5 mL/kg body weight bolus of iohexol injection was injected intravenously through the middle vein of the right elbow at a flow rate of 3.0 mL/s, followed by flushing with 20 mL of physiological saline. Arterial phase was obtained at 15–25 s, after intravenous injection of contrast. The detailed scanning parameters are listed in **Supplementary Table 2**.

Parameters	GE Revolution 256-slice spiral CT	Philips Brilliance iCT256	LianYing uCT780
Sample size	423	280	41
Tube voltage (kVp)	120	120	120
Tube current (mA)	automatic	automatic	automatic
Gathering matrix	512×512	512×512	512×512
Pitch	1.0	1.0	1.375
Layer thickness(mm)	1.25	1.25	2.5
Reconstruction thickness(mm)	0.625	0.625	0.625

Supplementary Table 3

The detailed Hyperparameters of seven machine learning models.

Models	Random Seed	Hyperparameters	Methods
DecisionTree	Seed(123)	ntree = 500,<br>mtry = sqrt(ncol(training_set) - 1),<br>importance = TRUE,<br>proximity = FALSE,<br>na.action = na.omit,<br>replace = TRUE,<br>sampsize = nrow(training_set),<br>nodesize = 1,<br>maxnodes = NULL	Grid Search, 10-Fold Cross-Validation
LRLASSO	Seed(123)	family = "LASSO",<br>alpha = alpha,<br>type.measure = "deviance",<br>nfolds = 10,<br>standardize = TRUE,<br>parallel = FALSE,<br>thresh = 1e-07,<br>maxit = 100000,<br>penalty.factor = rep(1, ncol(x_train)),<br>intercept = TRUE,<br>grouped = TRUE	Grid Search, 10-Fold Cross-Validation

KNN	Seed(123)	k = 15,<br>distance = 2,<br>kernel = "rectangular",<br>scale = TRUE,<br>use.all = TRUE,<br>prob = TRUE,<br>algorithm = "kd_tree",<br>tuneLength = 10,<br>cv_folds = 10	Grid Search, 10-Fold Cross-Validation
XGBoost	Seed(123)	booster = "gbtree",<br>objective = "binary:logistic",<br>eval_metric = "logloss",<br>max_depth = 6,<br>eta = 0.3,<br>gamma = 0,<br>min_child_weight = 1,<br>subsample = 0.8,<br>colsample_bytree = 0.8,<br>lambda = 1,<br>alpha = 0,<br>nthread = 4	Grid Search, 10-Fold Cross-Validation
SVM	Seed(123)	kernel = "radial",<br>cost = 1,<br>gamma = 0.5,<br>probability = TRUE,<br>scale = TRUE,<br>cross = 10,<br>cache_size = 100,<br>tolerance = 0.001,<br>epsilon = 0.1,<br>shrinking = TRUE,<br>class.weights = NULL	Grid Search, 10-Fold Cross-Validation
Bayes	Seed(123)	laplace = 1,<br>usekernel = TRUE,<br>adjust = 1,<br>fL = 0,<br>usekernal = TRUE,<br>prior = NULL,<br>threshold = 0.001,<br>eps = 0,<br>subset = NULL,<br>na.action = na.pass	Grid Search, 10-Fold Cross-Validation

NNet	Seed(123)	size = 5,<br>maxit = 200,<br>decay = 0.0001,<br>rang = 0.7,<br>skip = FALSE,<br>Hess = FALSE,<br>trace = TRUE,<br>MaxNWts = 1000,<br>abstol = 1.0e-4,<br>reltol = 1.0e-8,<br>learning_rate = 0.01,<br>activation = "logistic",<br>algorithm = "rprop+",<br>hidden_layers = 1,<br>batch_size = 10	Grid Search, 10-Fold Cross-Validation

Abbreviations: KNN, k-nearest neighbor; XGBoost, extreme gradient boosting classifiers; SVM, support vector machine; NNet, neural network.

Supplementary Table 4:

The specific number of features in the radiomics-feature filtering process.

Radiomics feature	Feature number	ICC≥0.8; Normalize with Z-score	T-test; Mann-Whitney *U*; Spearman's rank correlation > 0.9; mRMR	Top 15 radiomics features based on SHAP algorithm	Optimal number based on LASSO regression
Intra-aneurysm	851	787	222	15	9
Peri-aneurysmal 1mm	851	787	274	15	6
Intra-perianeurysmal 1mm	851	787	224	15	8

Abbreviations: LASSO, least absolute shrinkage and selection operator; ICC, Intraclass correlation coefficient; mRMR, Maximum Relevance Minimum Redundancy.

Supplementary Table 5:

The detailed calculation and construction process of Intra-aneurysm, Peri-aneurysmal 1mm, and Intra-perianeurysmal 1mm.

Intra-aneurysm	Feature Name	Coefficient values
IRSIAs	Intercept	-4.3261501
**A1**	original_ngtdm_Strength	-3.6151088
**A2**	wavelet.LHL_firstorder_90Percentile	-2.1115073
**A3**	wavelet.LHL_glszm_SizeZoneNonUniformityNormalized	-0.9633423
**A4**	wavelet.LHH_firstorder_Mean	1.2171887
**A5**	wavelet.HLL_ngtdm_Strength	-2.4590279
**A6**	wavelet.HLH_glcm_MaximumProbability	-4.5600655
**A7**	wavelet.LLL_firstorder_Minimum	8.1536772
**A8**	wavelet.LLL_firstorder_Skewness	3.3408759
**A9**	wavelet.LLL_ngtdm_Strength	-1.5220338
**IRSIAs** = -4.3261501+**A(1-9)***Coefficient values		
Peri-aneurysmal 1mm	Feature Name	Coefficient values
PRSIAs	Intercept	-3.1604755
**B1**	original_glszm_SmallAreaEmphasis	-1.3973156
**B2**	wavelet.HLL_ngtdm_Contrast	-2.5122929
**B3**	wavelet.HLH_glcm_ClusterShade	-0.5933798
**B4**	wavelet.HHL_glszm_ZonePercentage	-0.3383074
**B5**	wavelet.LLL_firstorder_10Percentile	5.5967023
**B6**	wavelet.LLL_ngtdm_Busyness	3.4220086
**PRSIAs** = -3.1604755+**B(1-6)***Coefficient values		
Intra-perianeurysmal 1mm	Feature Name	Coefficient values
IPRSIAs	Intercept	-7.7912672
**C1**	wavelet.HLH_glcm_ClusterShade	-0.1892142
**C2**	wavelet.HHH_glcm_JointEntropy	0.4170664
**C3**	wavelet.LLL_firstorder_10Percentile	7.3790410
**C4**	wavelet.LLL_firstorder_Skewness	8.4550964
**C5**	wavelet.LLL_glcm_Idn	0.4133328
**C6**	wavelet.LLL_ngtdm_Busyness	6.8022620
**C7**	wavelet.LLL_ngtdm_Coarseness	-1.0912509
**C8**	wavelet.LLL_ngtdm_Strength	-3.6273577
**IPRSIAs** = -7.7912672+**C(1-8)***Coefficient values		

CRS = 0.9696822+1.172528*IRSIAs+0.6046239*PRSIAs+0.4235017*IPRSIAs

Abbreviations: IAs, intracranial aneurysms; IRSIAs, the radiomics score for intra-aneurysm; PRSIAs, the radiomics score for peri-aneurysmal 1mm; IPRSIAs, the radiomics score for intra-perianeurysmal 1mm; CRS, the stacking radiomics score for intra-aneurysm, peri-aneurysmal 1mm and intra-perianeurysmal 1mm.

Supplementary Table 6:

The perfomance of 11 models for differentiating ruptured from unruptured IAs in training set.

Models	AUC(95%CI)	Accuracy	Precision	Recall	F1_Score	Brier_Score
Bayes	0.9150 (0.8872-0.9427)	0.8558	0.6283	0.7889	0.6995	0.1042
DecisionTree	0.8928 (0.8547-0.9309)	0.8983	0.7345	0.8646	0.7943	0.0853
KNN	0.9306 (0.9072-0.9540)	0.8676	0.6549	0.8132	0.7255	0.0933
LRLASSO	0.9228 (0.8966-0.9489)	0.8652	0.7917	0.6726	0.7273	0.0966
NNet	0.9289 (0.9046-0.9533)	0.8723	0.7434	0.7706	0.7568	0.0916
SVM	0.8767 (0.8305-0.9229)	0.8582	0.6106	0.8118	0.6970	0.1045
XGBoost	0.9853 (0.9767-0.9938)	0.9362	0.8258	0.9646	0.8898	0.0582
aMSLM	0.9516 (0.9334-0.9698)	0.8889	0.7345	0.8300	0.7793	0.0819
gMSLM	0.9444 (0.9237-0.9651)	0.8818	0.6991	0.8316	0.7596	0.0850
wMSLM	0.9589 (0.9426-0.9751)	0.8913	0.7522	0.8252	0.7870	0.0769
LRSLM	0.9880 (0.9794-0.9966)	0.9551	0.9292	0.9052	0.9170	0.0326

Abbreviations: KNN, k-nearest neighbor; LRLASSO, The logistic regression based on least absolute shrinkage and selection operator; NNet, neural network; SVM, support vector machine; XGBoost, extreme gradient boosting classifiers; DecisionTree, Decision Tree; aMSLM, the ensemble model was integrated for all machine learning prediction probability using arithmetic mean; gMSLM, the ensemble model was integrated for all machine learning prediction probability using geometric mean; wMSLM, the ensemble model was integrated for all machine learning prediction probability using weighted mean; LRSLM, the ensemble model was integrated for all machine learning prediction probability using logistic regression; AUC, area under the curve; CI, Confidence interval; IAs, Intracranial aneurysm.

Supplementary Table 7:

Subgroup analyses of different positions to explore the discriminatory efficacy of LRSLM in external validation set.

Aneurysm Location	N (ruptured/unruptured)	AUC	Sensitivity	Specificity
Posterior communicating artery	36 / 34	0.884	0.782	0.853
C7 segment of internal carotid artery	20 / 134	0.847	0.801	0.872
Anterior communicating artery	15 / 19	0.879	0.765	0.864
Middle cerebral artery	18 / 45	0.903	0.832	0.843

Abbreviations: LRSLM, the ensemble model was integrated for all machine learning prediction probability using logistic regression; AUC, area under the curve; IAs, Intracranial aneurysm

2. Supplementary Figures

Supplementary Figure 1:

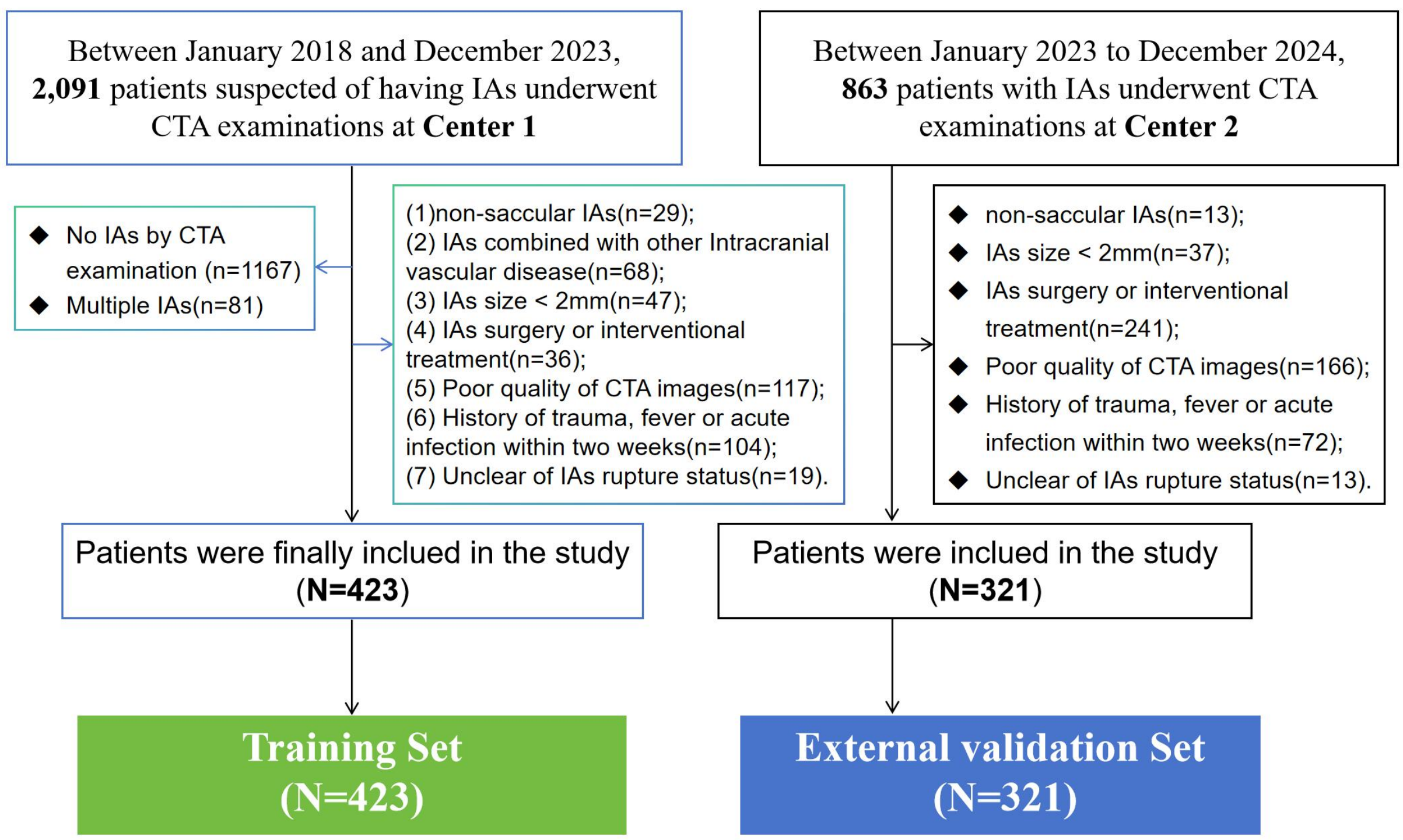

The flowchart of patient screening.

Supplementary Figure 2:

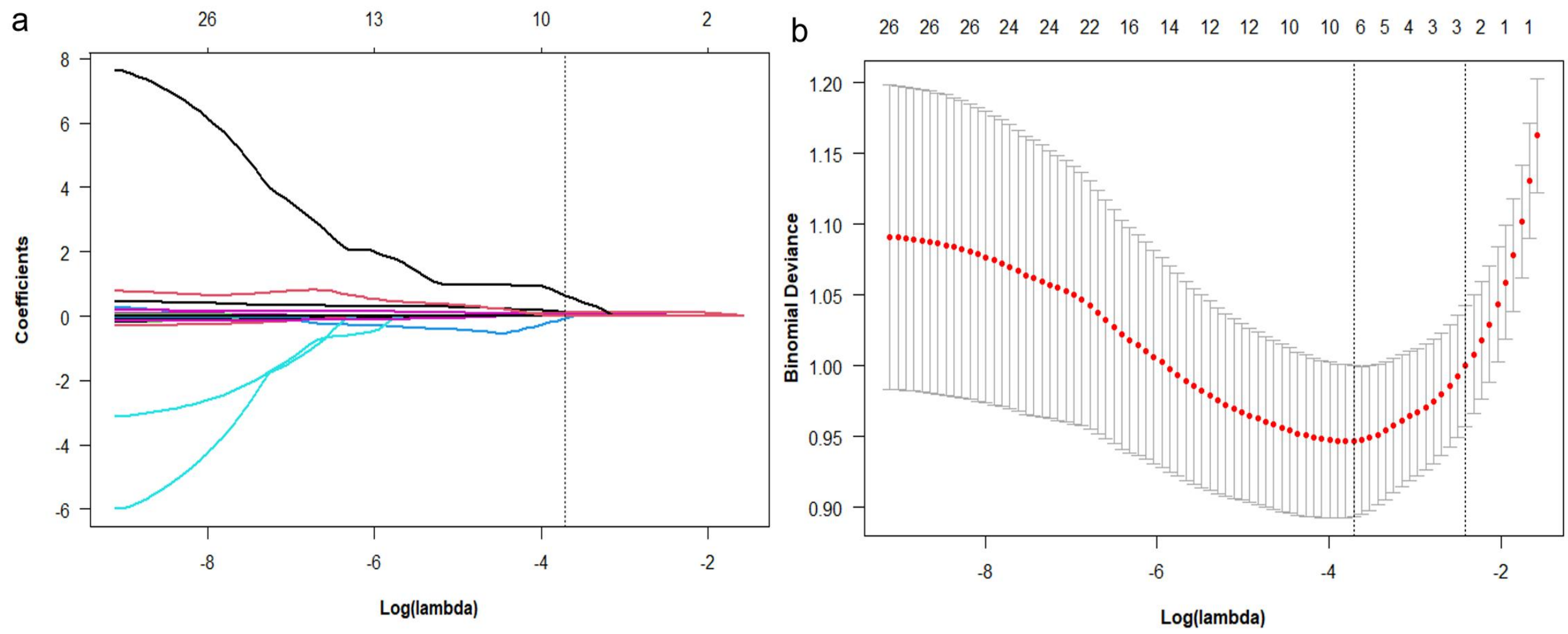

The LASSO regression (**a**) and ten fold cross validation (**b**) were used to screen the 27 serum biomarkers. Ultimately, six of the most important characteristics were selected using the the smallest Lambda (Lambda=0.0244287).

Supplementary Figure 3:

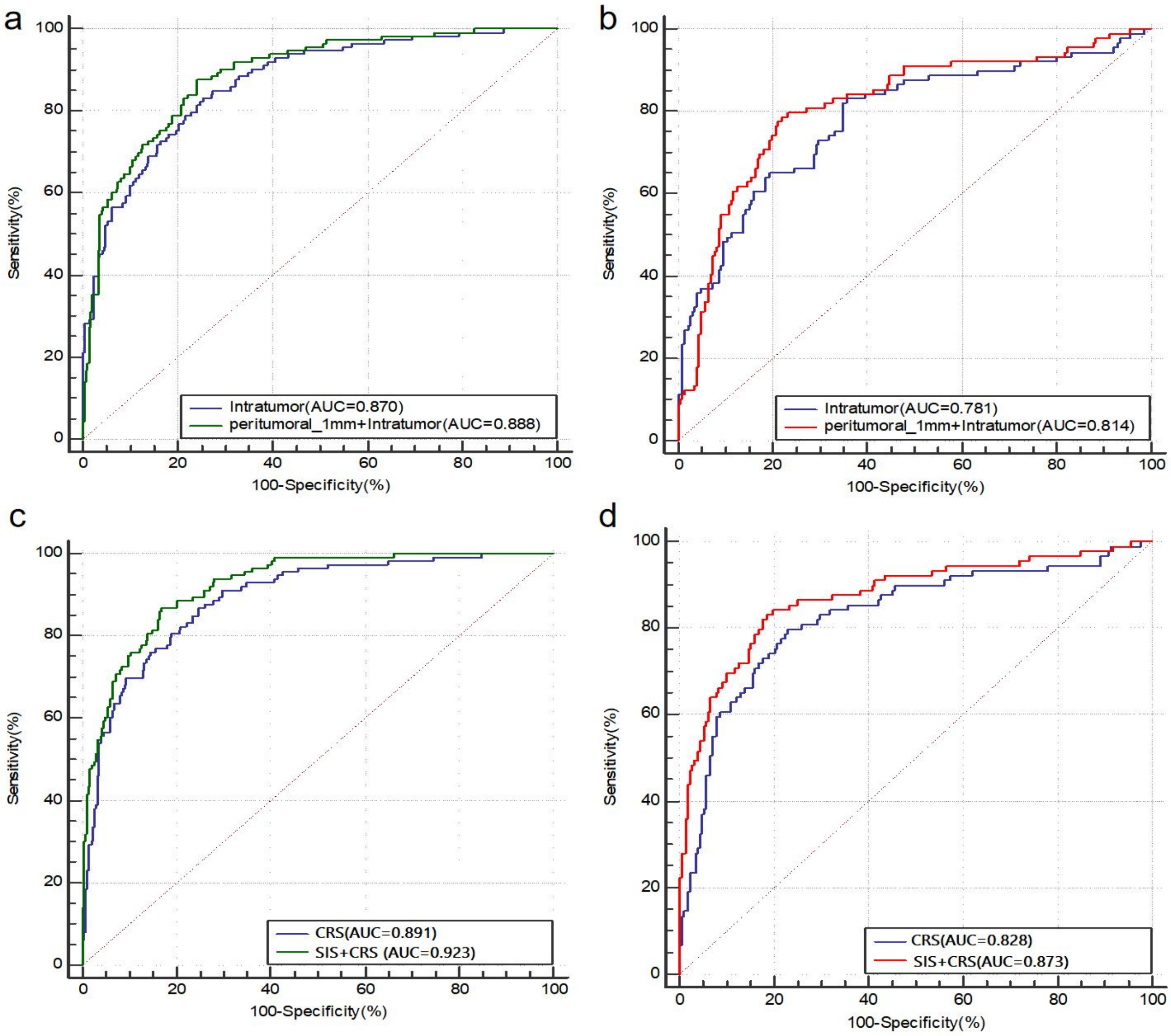

Adding peritumoral 1mm radiomics to intratumor of IAs could improve the AUC in training set (from 0.870 to 0.888; **a**) and external validation set (from 0.781 to 0.814; **b**). Adding SIS to CRS could improve the AUC in training set (from 0.891 to 0.923; **c**) and external validation set (from 0.828 to 0.873; **d**).

Supplementary Figure 4:

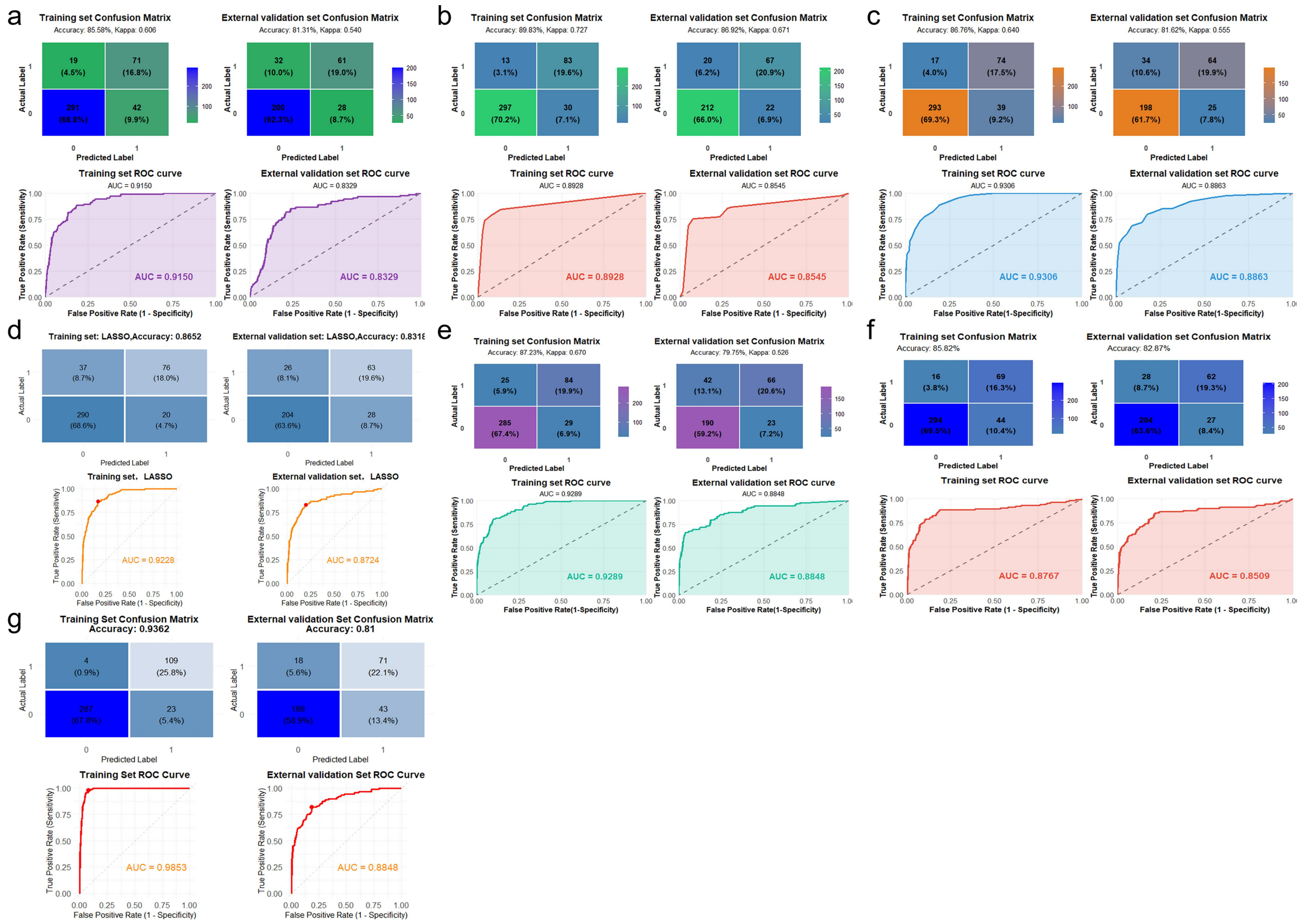

Confusion matrix and ROC curves of Bayes (**a**), DecisionTree (**b**), KNN (**c**), LRLASSO (**d**), NNet (**e**), SVM (**f**), Xgboost (**g**) for differentiating ruptured from unruptured IAs in training set and external validation set.

Abbreviations: ROC, receiver operating characteristic curve; KNN, k-nearest neighbor; LRLASSO, logistic regression using least absolute shrinkage and selection operator; NNet, neural network; SVM, support vector machine; Xgboost, extreme gradient boosting classifiers; AUC, area under the curve; CI, confidence interval; IAs, Intracranial aneurysm.

Supplementary Figure 5:

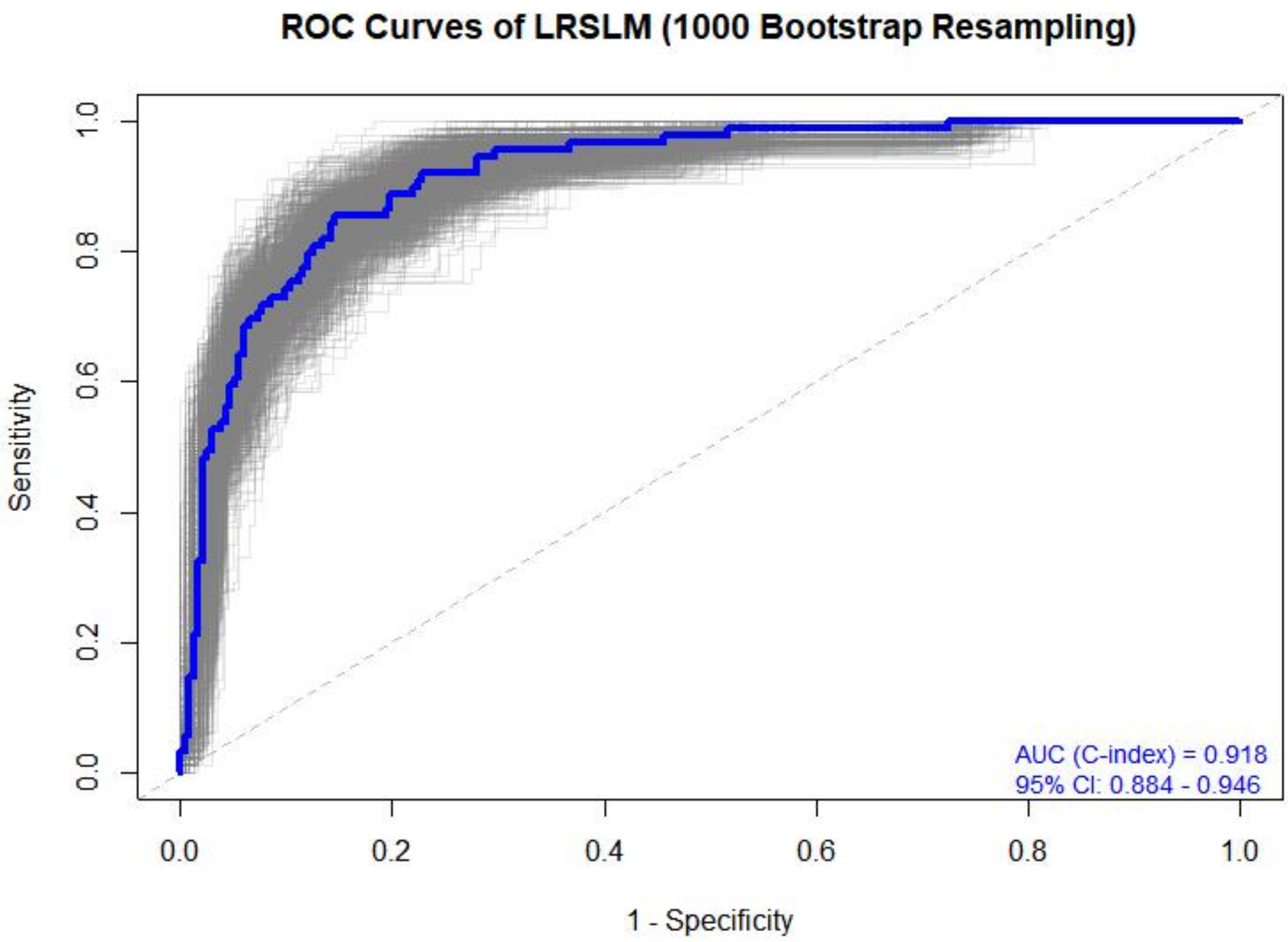

ROC curves of LRSLM (1000 Bootstrap Resampling). For the LRSLM, this 1000 Bootstrap Resampling yielded an AUC of 0.918 (95% CI: 0.884–0.946) on the external validation set.
